# Elucidation of dimethyl sulfide assimilation in soil bacteria expands the enzymatic landscape of terrestrial sulfur cycling

**DOI:** 10.1093/ismejo/wrag149

**Published:** 2026-06-13

**Authors:** Yasmeen Yousfi, Emilie Pateau, Nadia Perchat, Jean-Louis Petit, Murielle Jérôme, William Buchmann, Ekaterina Darii, Alain Perret

**Affiliations:** Génomique Métabolique, Genoscope, Institut François Jacob, CEA, CNRS, Univ Evry, Université Paris-Saclay, 91057 Evry, France; Génomique Métabolique, Genoscope, Institut François Jacob, CEA, CNRS, Univ Evry, Université Paris-Saclay, 91057 Evry, France; Génomique Métabolique, Genoscope, Institut François Jacob, CEA, CNRS, Univ Evry, Université Paris-Saclay, 91057 Evry, France; Génomique Métabolique, Genoscope, Institut François Jacob, CEA, CNRS, Univ Evry, Université Paris-Saclay, 91057 Evry, France; CNRS, LAMBE, Université Paris-Saclay, Univ Evry, CY Cergy Paris Université, 91025 Evry-Courcouronnes, France; CNRS, LAMBE, Université Paris-Saclay, Univ Evry, CY Cergy Paris Université, 91025 Evry-Courcouronnes, France; Génomique Métabolique, Genoscope, Institut François Jacob, CEA, CNRS, Univ Evry, Université Paris-Saclay, 91057 Evry, France; Génomique Métabolique, Genoscope, Institut François Jacob, CEA, CNRS, Univ Evry, Université Paris-Saclay, 91057 Evry, France

**Keywords:** bacterial metabolism, sulfur metabolism, LC–MS, GC–MS, metabolomics, enzyme kinetics, functional genomics, Featured image

## Abstract

Dimethyl sulfide (DMS) is a central volatile sulfur intermediate in the global sulfur cycle, traditionally associated with marine ecosystems. However, despite significant advances, important gaps remain in our understanding of its metabolism in terrestrial environments. Here, we elucidate a complete DMS catabolic pathway for sulfur utilization in the soil bacterium *Acinetobacter baylyi* ADP1 using an integrative approach combining targeted metabolomics, genetic knockouts, and *in vitro* enzyme assays. The pathway consists of a series of oxidation reactions catalyzed by two-component monooxygenases, including newly identified enzymes responsible for the sequential conversion of DMS to dimethyl sulfoxide and then dimethyl sulfone. These steps are followed by previously reported downstream enzymes that form methanesulfinate and methanesulfonate, ultimately yielding sulfite for cysteine biosynthesis. Functional redundancy and substrate promiscuity characterize these monooxygenases, all of which are powered by a single flavin reductase. A genomic survey revealed that this pathway is widespread among plant-associated and soil-dwelling Proteobacteria, including *Pseudomonas putida* and *Rhodococcus opacus*, which were experimentally confirmed to grow on DMS. Our findings reveal a widespread terrestrial DMS metabolic route that may represent a significant, yet previously unrecognized, component of the global sulfur cycle.

## Introduction

Dimethyl sulfide (DMS) is a major biogenic sulfur compound that strongly influences the global sulfur cycle. It is primarily produced from the degradation of dimethylsulfoniopropionate (DMSP) by marine microalgae and bacteria [1]. Global DMS emissions range from 13 to 37 Tg of S per year [2, 3] and represent 50%–60% of the natural sulfur flux to the atmosphere. Released DMS is quickly oxidized into dimethylsulfoxide (DMSO), which then converts to sulfate aerosols. These aerosols play a role in cloud formation and albedo regulation, thereby affecting Earth’s climate [4]. These sulfates then return to terrestrial systems through acid rain deposition [5].

**Figure 1:**
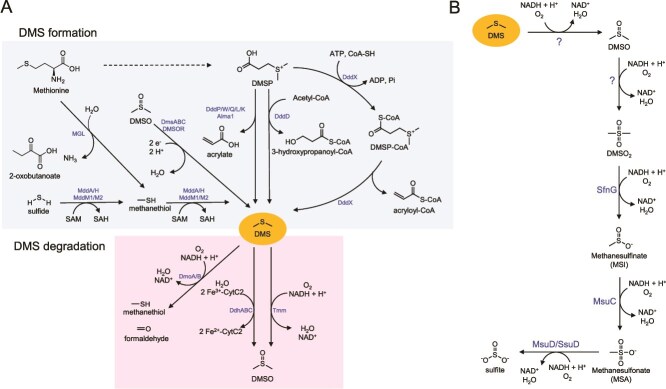
Overview of DMS metabolism. (A) Formation and degradation of DMS in various organisms. Upper panel, enzymatic reactions leading to the formation of DMS. The DMSP lyases DddP, DddW, DddQ, DddL, DddK, and Alma1 converts DMSP to acrylate and DMS. DddD converts DMSP to 3-hydroxypropanoyl-CoA and DMS, using acetyl-CoA as a CoA donor. DddX converts DMSP to DMSP-CoA, then to acryloyl-CoA and DMS, with ATP and CoA as co-substrates. The DMSO reductases DmsABC and DMSOR reduce DMSO to DMS. The S-methyltransferases MddA, MddH, MddM1, and MddM2 can methylate hydrogen sulfide into methanethiol and then into DMS, using SAM as a co-substrate. The MGL forms methanethiol, which can be converted into DMS. The dotted line summarizes the various enzymatic steps that convert methionine into DMSP. Lower panel, enzymatic reactions using DMS as a substrate. The two-component monooxygenase system DmoA/B catalyzes the oxidation of DMS into DMSO. The DMS dehydrogenase DdhABC is a molybdoenzyme complex that oxidizes DMS into DMSO. Trimethylamine monooxygenase (Tmm) is a flavin-containing monooxygenase that converts DMS into DMSO. (B) Proposed DMS catabolic pathway in *Pseudomonas*. Identified genes are indicated. MGL, methionine γ-lyase; SAM, S-adenosylmethionine.

DMS is formed via multiple microbial pathways (Fig. 1A). The best-studied route involves DMSP cleavage into DMS and acrylate by DMSP lyases, including DddP [6], DddW [7], DddQ [8], DddL [9], DddK [10], and the algal enzyme Alma1 [11]. Other enzymes such as DddX and DddD cleave DMSP into DMS and Coenzyme A (CoA) derivatives, such as acryloyl-CoA and 3-hydroxypropionyl-CoA [12, 13]. DMS is also produced via DMSO reduction by molybdenum-containing reductases in organisms such as *Escherichia coli* (DmsABC) and *Rhodobacter sphaeroides* [14, 15]. In soils, methanethiol methylation by the enzymes MddA, MddH, MddM1, and MddM2 represents an additional source [16–19]. These enzymes also catalyze the methylation of H_2_S into methanethiol, thereby providing a route for H_2_S to serve as a precursor of DMS [17]. In terrestrial environments, methionine gamma-lyase generates methanethiol, which can then undergo methylation to form DMS.

Bacterial DMS degradation is less characterized, although marine microbes oxidize 80% of DMS produced in the marine environment [20]. Depending on the organism, DMS may function as a source of carbon, energy, or sulfur. Two major degradation mechanisms have been described: oxidation to DMSO and cleavage to methanethiol and formaldehyde.

Oxidation to DMSO represents the primary sink for DMS in surface ocean waters [21]. Tmm, a trimethylamine monooxygenase widespread in the SAR11 clade and the marine *Roseobacter* lineages, converts DMS to DMSO, which appears to be an end-product that is eventually excreted [22, 23]. Tmm is a class B bacterial flavin-containing monooxygenase that binds flavin adenine dinucleotide (FAD) and uses reduced nicotinamide adenine dinucleotide phosphate (NADPH) [24]. Given its widespread distribution in marine bacteria, Tmm is likely a major contributor to global DMS turnover. Another enzyme, the DMS dehydrogenase DdhABC, has been characterized in *Rhodovulum sulfidophilum* [25]. This periplasmic molybdoenzyme complex channels electrons from DMS oxidation into the photosynthetic electron transport chain. DMS oxidation has also been described in methylotrophic and sulfur-oxidizing bacteria, such as *Hyphomicrobium sulfonivorans* and *Thiobacillus thioparus* [20, 26]. In *H. sulfonivorans*, this reaction is catalyzed by a two-component monooxygenase system: a flavin mononucleotide (FMNH_2_)-dependent monooxygenase (DmoA) coupled to a flavin reductase (DmoB), both encoded by neighboring genes [20]. *H. sulfonivorans* can use DMS as a source of carbon, energy, and/or sulfur [27], assimilating part of the resulting formaldehyde via the serine cycle, and oxidizing the rest to CO_2_. In *T. thioparus*, where DMS can serve as a carbon and energy source, oxidation of DMS to DMSO has been observed, although the enzymes responsible have not yet been identified. In contrast, in soil- and plant-associated habitats, DMS degradation can follow a specialized oxidative route focused on sulfur utilization. In *Rhodococcus* sp. strain SY1, DMS undergoes sequential oxidation to DMSO, dimethylsulfone (DMSO_2_), and methanesulfonate (MSA), yielding methane and sulfite. Sulfate production was presumed to result from subsequent sulfite oxidation [28]. Although DMS oxidation genes remain unidentified in *R.* strain SY1, the multicomponent system DsoABCDEF, catalyzing stepwise oxidation to DMSO and DMSO_2_, has been genetically characterized in *Acinetobacter sp*. 20B [29]. Similar to phenol hydroxylase [30], the enzyme comprises a FAD- and [2Fe-2S]-containing reductase, an activator protein, and an oxygenase component that harbors the active site and a dinuclear iron center [31].

*Pseudomonas* species, such as *Pseudomonas putida* DS1 and *Pseudomonas aeruginosa* PAO1, also oxidize DMS to DMSO and DMSO_2_ [32, 33] to ultimately produce sulfite for cysteine biosynthesis (Fig. 1B). This process involves two-component monooxygenase systems encoded by the *ssuEADCBF* operon in *P. putida* [32] and the *msuEDC* operon in *P. aeruginosa* [34]. In these organisms, the reductases SsuE and MsuE supply FMNH_2_ to the monooxygenases SsuD and MsuD that cleave MSA to sulfite and formaldehyde [32, 34, 35]. *Pseudomonas* also utilizes SfnFG, another two-component monooxygenase system, to oxidize DMSO_2_ to methanesulfinate (MSI) [36, 37], followed by conversion of MSI to MSA by the monooxygenase MsuC [38]. However, the genes responsible for the initial oxidation of DMS to DMSO_2_ in *Pseudomonas* remain unknown because the DsoABCDEF system of *Acinetobacter* sp. 20B is absent from the genomes of *P. putida* KT2440 [39] and *P. aeruginosa* PA01 [40]. This major gap limits our understanding of DMS turnover in terrestrial environments.

*Acinetobacter baylyi* ADP1 (ADP1) is a ubiquitous soil bacterium whose genome shares similarity with those of *P. aeruginosa* and *P. putida* [41]. ADP1 can utilize a broad spectrum of compounds as carbon and energy sources [42] and metabolize diverse sulfur compounds [43], including DMS. It carries homologs of the genes encoding the enzymes that catalyze the last three steps of the pathway proposed in *Pseudomonas* (Fig. 1B). Furthermore, ADP1 is an ideal model for studying bacterial metabolism because of the ease with which it can be genetically modified [44].

In this study, we provide a comprehensive characterization of DMS metabolism in ADP1. Using a metabolomics-based strategy, we show that ADP1 degrades DMS through a pathway similar to that described in *Pseudomonas*, sequentially producing DMSO_2_, MSI, MSA, and sulfite (Fig. 1B). Through targeted mutant phenotyping, we identified the terminal genes responsible for converting DMSO_2_ to sulfite, consistent with their homology to known genes in *Pseudomonas*. Candidate enzymes for the two upstream oxidation steps were identified via activity-based screening and kinetically characterized. Deletion mutants were then used to assess the *in vivo* contribution of each enzyme. Finally, phylogenetic analysis revealed that the key genes of this DMS catabolic pathway are broadly distributed among *Pseudomonadota*, suggesting that this terrestrial DMS degradation route is more widespread than previously recognized and may represent an important sink for reduced sulfur in soil ecosystems.

## Materials and methods

### Materials and reagents

All chemicals were purchased from Sigma-Aldrich (St. Louis, USA) and Enamine (Kyiv, Ukraine). Molecular biology reagents and kits were from Invitrogen (Carlsbad, USA). Oligonucleotides were from Sigma-Aldrich.

### Strains and medium

ADP1 (DSM 24193) was provided by Dr Nicholas Ornston (Yale University). All strains were routinely grown in aerated culture vessels on MP minimal medium (10.3 mM Na_2_HPO_4_; 4.76 mM KH_2_PO_4_) supplemented with 20 mM NH_4_Cl, 7.5 μM FeCl_3_, 2 mM MgCl_2_, 25 mM succinate as the carbon source, and 2 mM MgSO_4_ or 2 mM of the desired sulfur source—except for DMS, which was added at an initial concentration of 10 mM to compensate for its high volatility and ensure its availability during aerobic growth.

### Metabolome preparation

For liquid chromatography–mass spectrometry (LC–MS) metabolomics, overnight cultures that had reached stationary phase were diluted to an OD_600_ of 0.1 in a 24 deep-well plate (UNIPLATE microplates; Whatman) containing 5 ml of fresh medium, and further grown until reaching an OD_600_ between 0.6 and 0.8. Metabolomes were then extracted as described previously [45]. Dried metabolomes were suspended in 100 μl water and filtered on 0.22 μm polytetrafluoroethylene (AcroPrep Advance, Pall).

### Mass spectrometry analyses

#### GC-–MS/MS analysis

Gas chromatography-tandem mass spectrometry (GC–MS/MS) analyses were performed on a 7010B triple quadrupole coupled to a 7890B GC system (Agilent Technologies). Liquid injections were performed using an automatic liquid sampler. The injection volume was 1 μl. The septum purge flow rate was set to 3.0 ml min^−1^. The injector operated in split mode with a split ratio of 10:1 and a split flow of 10 ml min^−1^. The injection temperature was set to 250°C. The instrument was equipped with a 30 m × 0.25 mm × 0.25 μm HP-5MS column (Agilent). Helium was used as the carrier gas under constant pressure mode at 34.976 psi. The oven was held at 75°C for 1 min after injection, then ramped at 15°C min^−1^ to 170°C with no hold time. The ionization source temperature was set to 230°C and the transfer line to 250°C. Ionization was carried out by electron impact at 70 eV. Data were acquired in full-scan mode over m/z 35–400. Multiple reaction monitoring (MRM) experiments were acquired using N2 as the collision gas, with a dwell time of 50 ms per transition. Collision energies were optimized for each transition from standard compounds. The complete list of transitions and parameters is provided in Supplementary Table S1. Data were processed using MassHunter B.08.00.

#### LC-–MS analysis

Analyses were conducted using a Dionex Ultimate 3000 Rapid Separation LC (Thermo Fisher Scientific) using a C18 column (4.6 × 150 mm; 5 μm beads; Waters, USA) coupled to a hybrid triple quadrupole linear ion trap mass spectrometer (QTRAP 5500 from ABSciex) equipped with a heated electrospray ionization source. Chromatographic separation was achieved on a X-Bridge C18 column at 40°C as follows: a mobile phase gradient was used with a flow rate of 0.3 ml min^−1^ in which mobile phase A consisted of 0.1% (vol/vol) formic acid and mobile phase B consisted of methanol. The gradient started at 95% A for 2 min, followed by a linear gradient at 90% B for 14 min and remained 3 min at 90% B. The system returned to the initial solvent composition in 2 min and re-equilibrated under these conditions for 12 min. The autosampler was kept at 4°C and 5 μl was injected. MS analyses were conducted with the following parameters: ion source 4500 V (positive mode) or −4500 V (negative mode), curtain gas 20 psi, temperature 400°C, gas 1—40 psi, gas 2—60 psi. Data were acquired in MRM with Q1 and Q3 at unit resolution. Fragmentation parameters were optimized for each transition (Supplementary Table S2). Data were processed using Analyst 1.6.2 (ABSciex). For both GC and LC MRM methods, when feasible, two transitions were monitored per analyte; only one transition (quantifier) was used for molecular quantitation.

### Cloning, expression, and purification of the recombinant enzymes

The genes of interest were retrieved from an existing in-house library of pET22b(+)-derived expression plasmids previously established for the recombinant production of cytosolic enzymes in *E. coli* [46]. Each expression plasmid was transformed into competent *E. coli* BL21 (DE3) strain (Invitrogen, USA). Protein purification was performed using a preparative chromatography system (Äkta Pure; Cytiva). A fully automated two-step method was set up for each protein in which a HisTrap FF 1 ml (Cytiva) column was used in the first purification step. The eluted peak was redirected on a 5 ml HiTrap desalting column (Cytiva) and collected in 50 mM Tris-HCl (pH 8.0), 50 mM NaCl, and 15% glycerol.

### Enzymatic assays

The Standard Reaction Mixture (SRM) contained 25 mM Tris-HCl pH 7.5, 100 mM NaCl, 1 μM FMN, and 1 mM NADH. Unless otherwise specified, all assays were performed in 500 μl of SRM.

Monooxygenase activity screening: Monooxygenase activity was monitored by LC–MS/MS and conducted in SRM, with 500 μM of the desired substrate for 5 min. Monooxygenase concentration was fixed at 0.2 μM in all assays. MsuE concentration was 0.6 μM for group C enzymes and 0.2 μM for group D enzymes (Supplementary Table S3). The reactions were stopped with 1% (vol/vol) trifluoroacetic acid and neutralized with K_2_CO_3_ before LC–MS/MS analysis.

Determination of specific activities: activities were monitored by GC–MS/MS and conducted in SRM with 400 μM of the desired substrate over a 3-min time course, using 1-min sampling intervals. Accumulation of DMSO and DMSO_2_ with time was linear up to 3 min. Monooxygenase concentration was fixed at 2 μM in all assays. MsuE concentration was 6 μM for group C enzymes and 2 μM for group D enzymes. Reactions were stopped with 1% (vol/vol) trifluoroacetic acid. Enzymatic reactions were extracted with two volumes of CH_2_Cl_2_ before injection.

The kinetic parameters of MsuE were determined by monitoring the absorbance of NADH at 340 nm as a function of time. Experiments were conducted in 500 μl of 25 mM Tris-HCl pH 7.5, 100 mM NaCl. The kinetic parameters of SsuD were determined using a spectrophotometric assay adapted from Somai *et al*. [47]. Briefly, enzymatic reactions were conducted in SRM with varying concentrations of MSA. The reactions were initiated by the addition of 500 μM NADH, incubated for 0, 1, 2, and 3 min and stopped with 2 M urea.

The kinetic parameters of MsuD and MsuC were determined by LC–MS/MS. Enzymatic reactions were conducted in SRM with varying concentrations of DMSO_2_ or MSI for 0, 1, 2, or 3 min. Accumulation of MSI and MSA with time was linear up to 3 min. For MsuD, reactions contained 0.6 μM MsuE and 0.2 μM monooxygenase. For MsuC, reactions contained 0.2 μM MsuE and 0.2 μM monooxygenase. The reactions were stopped with trifluoroacetic acid and neutralized with K_2_CO_3_ before LC–MS/MS analysis.

The kinetic parameters of Dms1, Dms2, and Dms3 were determined by GC–MS/MS. Enzymatic reactions were conducted in SRM with varying concentrations of DMS or DMSO for 0, 1, 2, or 3 min. Accumulation of DMSO and DMSO_2_ with time was linear up to 3 min. Monooxygenase concentration was fixed at 2 μM in all assays. MsuE concentration was 6 μM for group C enzymes and 2 μM for group D enzymes. Reactions were stopped with 1% (vol/vol) trifluoroacetic acid. Enzymatic reactions were extracted with two volumes of CH_2_Cl_2_ before injection.

All experiments were conducted at 25°C.

### Construction of deletion mutants

The construction of single-gene deletion mutants in ADP1 was already described [48]. The double mutant *Δdms1–dms2* was obtained by simultaneously deleting both target genes using a single kanamycin resistance cassette (Kan^R^). As the two genes were located adjacently in the genome, the entire region encompassing both open reading frames was replaced in a single step. The triple mutant *Δdms1–dms2–dms3* was constructed from *Δdms1–dms2* by replacing *dms3* with an apramycin resistance cassette (Apra^R^). The knockout strains were selected on minimal medium agar supplemented with 30 μg min^−1^ kanamycin or kanamycin plus apramycin. The Oligonucleotide primers used are listed in Supplementary Table S4.

### Expression analysis by reverse transcriptase quantitative PCR (RT-qPCR)

The mRNA levels of *msuE, dms1, dms2, dms3, msuD*, and *ssuD* were determined by reverse transcriptase quantitative polymerase chain reaction (RT-qPCR) with *rpoB* as internal standard for expression normalization. Cultures were performed in MP minimal medium supplemented with 20 mM NH_4_Cl, 7.5 μM FeCl_3_, 2 mM MgCl_2_, 25 mM succinate as the carbon source, and 2 mM MgSO_4_ or 10 mM DMS as the sulfur source. Cells were cultured in triplicate and harvested in exponential phase. Total RNA was extracted using the RNeasy Plus Universal Mini Kit (Qiagen, Hilden, Germany) following the manufacturer’s protocols. DNA was removed from the samples using the TURBO DNase Kit (Invitrogen) and RNA was then treated with RNA Clean and Concentrator-5 (Zymo Research, USA), and assayed with RNA 6000 Picokit on a Bioanalyser (Agilent Technologies). cDNA was generated using the High-Capacity cDNA Reverse Transcription Kit (Applied Biosystems) and its concentration was determined using Nanodrop (Thermo Scientific). Quantitative real-time PCR experiments were performed for two technical replicates, each analyzed for four template dilutions using the KAPA SYBR FAST Kit (Roche). Primer pairs used for amplification of *msuE, dms1, dms2, dms3, msuD, ssuD*, and *rpoB* are listed in Supplementary Table S4 (efficiency above 95%). cDNA was used for quantitative PCR with a Stratagene MX3005P (Agilent Technologies) using PowerTrack SYBR Green Master Mix (Applied Biosystems).

### Phylogenomic and rRNA gene analyses

The three ADP1 sequences (SsuD, Dms2, and MsuD) were each used as reference proteins in our CAESAR pipeline (CAndidate Enzyme SeARch; https://github.com/labgem/CAESAR). Candidate homologs were selected based on ≥50% identity and ≥80% coverage relative to the reference sequences using Basic Local Alignment Search Tool Protein (BLASTP) against the National Center for Biotechnology Information (NCBI) database. Only genomes containing homologs of all three reference genes were retained. For these strains, we then retrieved their clearly annotated 16S rRNA gene sequences—namely, sequences explicitly labeled as “16S rRNA” and ranging from 1300 to 1600 nucleotides in length in the NCBI database. Phylogenetic analysis was performed using Multiple Alignment using Fast Fourier Transform (MAFFT) [49] for multiple sequence alignment, QuickTree [50] for tree building, and Interactive Tree Of Life (iTOL) [51] for tree rendering.

### Environmental metagenome analysis

Environmental metagenomic analyses were conducted as previously described [18, 52]. Dms1, Dms2, MsuD, SsuD, and RecA protein sequences from organisms able to use DMS as a sulfur source (Supplementary Table S5) were used as queries to search metagenomic datasets through the Integrated Microbial Genomes and Microbiomes (IMG/M) platform [52]. Homologs were identified in metagenomes from different environments (Supplementary Table S6) using BLASTP implemented in IMG/M [52], applying an e-value cutoff of 10^−30^ and a minimum identity of 30% relative to the reference sequences [18, 52]. The abundance of Dms1, Dms2, MsuD, and SsuD homologs was estimated by normalizing the numbers of unique hits to the number of unique RecA sequences, assuming a single copy of *recA* per bacterial genome.

## Results

### Validation of the DMS catabolic pathway in ADP1

**Figure 2:**
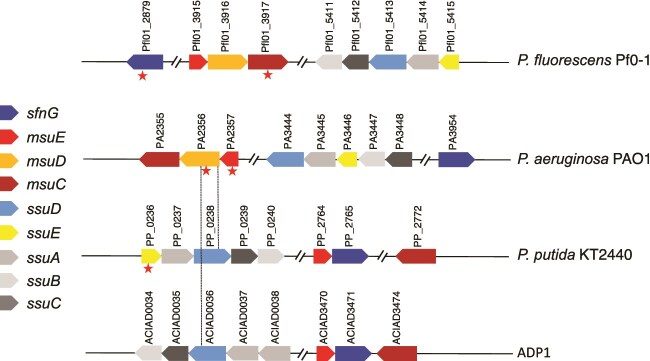
Genetic organization of the known genes involved in DMS assimilation in *Pseudomonas* and comparison with ADP1. Gene co-localization was examined using the MicroScope platform [53]. Stars indicate genes whose function has been experimentally validated in the corresponding organism [34, 37, 38, 54]. Genes *ssuA, ssuB,* and *ssuC* encode the components of an ABC-type transporter system. Gene *ssuE* encodes a flavin reductase. Gene *msuD* is absent from the genomes of *Pseudomonas putida* and ADP1. Dotted lines highlight genes in these organisms whose encoded proteins show the highest sequence identity to *Pseudomonas aeruginosa* PA2356 (MsuD). The symbol “//” indicates an interruption of >5 kb within the gene cluster.

Given the phylogenetic proximity between *Pseudomonas* and *Acinetobacter* within *Pseudomonadales*, we hypothesized that ADP1 metabolizes DMSO_2_ via the same pathway (Fig. 1B). We therefore searched the ADP1 genome for homologs of the three enzymes mediating DMSO_2_ utilization in *Pseudomonas* and examined their genomic context (Fig. 2). SfnG, characterized in *P. putida* SF1 [36], converts DMSO_2_ to MSI and shares 80% amino acid identity with ACIAD3471 (annotated as MsuD) in ADP1 (95% with PP_2765 in *P. putida* KT2440). MsuC, identified in *Pseudomonas fluorescens* Pf0-1 [38], oxidizes MSI to MSA and exhibits 55% identity with ACIAD3474 (78% with PP_2772 from KT2440). Finally, SsuD, described in *P. putida* DS1 [32], catalyzes MSA desulfonation and shares 67% identity with ACIAD0036 in ADP1 (97% with PP_0238 from KT2440). We therefore propose to name ACIAD3471, ACIAD3474, and ACIAD0036 as MsuD, MsuC, and SsuD, respectively (Supplementary Table S7).

**Figure 3:**
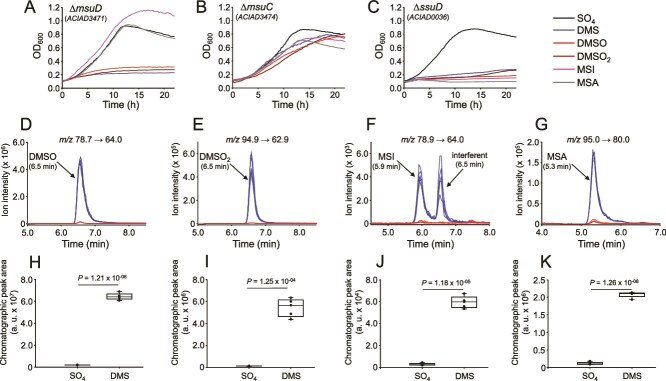
Validation of the DMS catabolic pathway in *Acinetobacter baylyi* ADP1. (A–C) Growth phenotyping of ∆*msuD*, ∆*msuC*, and ∆*ssuD* on various sulfur sources. Absorbance at 600 nm was recorded by an automated growth curve analysis system (Bioscreen-C; Thermo Fisher Scientific). Data correspond to the average of three biological replicates. (D–G) Extracted ion chromatograms of DMSO, DMSO_2_, MSI, and MSA, respectively, from intracellular metabolomes of cells grown with DMS (blue) or sulfate (red) as the sole sulfur source. (H–K) Relative levels of DMSO, DMSO_2_, MSI, and MSA, respectively, in metabolomes of cells grown with DMS or sulfate. LC–MS/MS analyses were performed on a QTRAP 5500 mass spectrometer using a MRM-based approach. DMSO and DMSO_2_ were detected in the positive ionization mode; MSI and MSA were detected in the negative ionization mode. The monitored mass transitions were: DMSO, m/z 78.7 → 64.0; DMSO_2_, m/z 94.9 → 62.9; MSI, m/z 78.9 → 64.0; and MSA, m/z 95.0 → 80.0. Values correspond to automated peak integration. Metabolites were quantified from five independent cultures per strain. *P*-values were determined by Welch’s *t*-test.

To evaluate the involvement of these candidate enzymes in sulfur utilization from DMS, we examined the growth of the deletion mutants ∆*msuD*, ∆*msuC*, and ∆*ssuD* on various sulfur sources (Fig. 3A–C). ∆*msuD* showed severely impaired growth on DMS, DMSO, and DMSO_2_, consistent with the essential role of this gene in converting DMSO_2_ to MSI, as reported in other organisms [36, 37]. ∆*msuC* showed no significant defect on these sulfur sources, in agreement with reports suggesting that MSI oxidation to MSA can proceed independently of MsuC, as a reaction mediated by FMNH_2_ [38]. Finally, ∆*ssuD* displayed impaired growth on all sulfur sources, supporting its role in the terminal desulfonation step.

These phenotypic data support the three last reactions (catalyzed by SfnG, MsuC, and MsuD/SsuD; Fig. 1B) and the functional involvement of the identified homologs in ADP1 DMSO_2_ metabolism.

To obtain definitive evidence for the existence of this pathway, we performed targeted metabolomics on cells grown on DMS as the sole sulfur source. The expected intermediates (DMSO, DMSO_2_, MSI, and MSA) were detected in DMS-grown cells but were undetectable in sulfate-grown controls (Fig. 3). The peak corresponding to MSI (RT 5.9 min) was confirmed by spiking samples with an MSI reference compound (Supplementary Fig. S1).

### Activity-based screening for enzymes involved in DMS and DMSO oxidation

Previous studies have provided evidence about the type of enzymes catalyzing the oxidation of DMS to DMSO and DMSO_2_. In *Rhodococcus* sp. strain SY1, a dibenzothiophene (DBT)-desulfurizing bacterium, DMS is proposed to undergo sequential oxidation to DMSO and then to DMSO_2_ [28]. DBT desulfurization in *Rhodococcus* IGTS8 involves two initial oxidation steps (oxidation to sulfoxide and sulfone) catalyzed by DszC, a monooxygenase that receives reduced flavin from the NADH/FMN reductase DszD [55–57]. The mechanistic similarity between DBT and DMS oxidation suggests that DMS conversion to DMSO_2_ in *Pseudomonas* and ADP1 may likewise rely on flavin-dependent monooxygenases.

To identify ADP1 enzymes potentially involved in DMS oxidation, we surveyed monooxygenase genes annotated in the MicroScope genome annotation platform ([53]; https://mage.genoscope.cns.fr/microscope). Among 34 genes, we selected 16 linked to sulfur metabolism (Supplementary Table S3). Because these putative monooxygenases are flavin-dependent, we identified ACIAD3470—annotated as MsuE and sharing 56% identity with the biochemically characterized flavin reductase MsuE (Pfl01_3915) from *P. fluorescens* Pf0-1—as the likely flavin reductase partner [58]. All genes were cloned for overexpression in *E. coli* and the corresponding proteins (except ACIAD2064 and ACIAD2065, produced as inclusion bodies) were purified for DMS oxidation assays.

### Kinetic properties of MsuE_ADP1_

In *P. fluorescens* Pf0-1, MsuE is a NADH:FMN oxidoreductase that provides FMNH_2_ for monooxygenase activity [37]. MsuE_ADP1_ activity was confirmed by monitoring NADH oxidation in the presence of FMN. Because monooxygenases are divided into two groups (C and D) based on whether they used reduced FMN or FAD [59–64], we tested the flavin specificity of MsuE_ADP1_. MsuE_ADP1_ accepted both FMN and FAD with similar catalytic efficiency (1.4 × 10^6^ and 5.7 × 10^5^ s^−1^ M^−1^, respectively, Table 1) and was also active with NADPH, though 50-fold less efficiently than with NADH (as indicated by the ratio between *k_cat_/K_m_* NADH and *k*_cat_/*K*_m_ NADPH). Whether these enzymatic properties are specific to MsuE_ADP1_ remains to be determined.

**Table 1:** Kinetic properties of MsuE.

Enzyme	Substrate	*K*_m_ (μM)	*k*_cat_ (s ^ −1^ )	*k*_cat_ /*K*_m_ (s ^ −1^ M ^ −1^ )
MsuE	FMN^a^	8.6 ± 5.0	11.9 ± 1.6	1.4 × 10^6^
	NADH^b^	24.8 ± 3.9	11.9 ± 0.6	4.8 × 10^5^
	NADPH^b^	267.4 ± 43.7	2.4 ± 0.2	9.0 × 10^3^
	FAD^a^	19.5 ± 9.4	11.2 ± 1.7	5.7 × 10^5^

Values correspond to the average of three replicates.

^a^NADH concentration was 450 μM.

^b^FMN concentration was 150 μM.

### Experimental setup for activity-based screening

Flavin transfer in two-component system oxygenases can occur either via free flavin diffusion [65, 66] or transient reductase-oxygenase complexes [67, 68]. The 14 candidate enzymes were classified into 2 different groups based on the flavoprotein monooxygenase classification system [69]. Nine enzymes were assigned to group C (TIM barrel fold) and five to group D (acyl-CoA dehydrogenase fold) (Supplementary Table S3).

Due to structural differences between the two groups, we hypothesized that the mode of interaction with MsuE_ADP1_ may differ, requiring distinct MsuE:monooxygenase molar ratios for optimal activity. SsuD_ADP1_ (group C) exhibited the anticipated MSA monooxygenase activity and showed maximal activity at a 3:1 ratio (Supplementary Fig. S2A), as previously observed for SsuD*_E. coli_* [59]. Preliminary assays showed that ACIAD0428 (group D) oxidized DMSO to DMSO_2_. It reached maximal activity at a 1:1 ratio (Supplementary Fig. S2B). Based on these results, subsequent assays used a 3:1 ratio for group C enzymes and a 1:1 ratio for group D enzymes.

### Identification of genes involved in DMSO and DMSO_2_ formation

To identify monooxygenases active with DMS and DMSO, each enzyme was assayed *in vitro* on both substrates in the presence of MsuE. Reactions were conducted over a 5-min period, and product formation was analyzed by LC–MS/MS. DMSO was detected in all reactions with DMS, including controls with MsuE alone, indicating FMNH_2_-mediated oxidation rather than monooxygenase-specific activity [38]. Because different MsuE concentrations were used for group C (0.6 μM) and group D (0.2 μM) enzymes, which resulted in different detection thresholds, the groups were analyzed separately.

**Figure 4:**
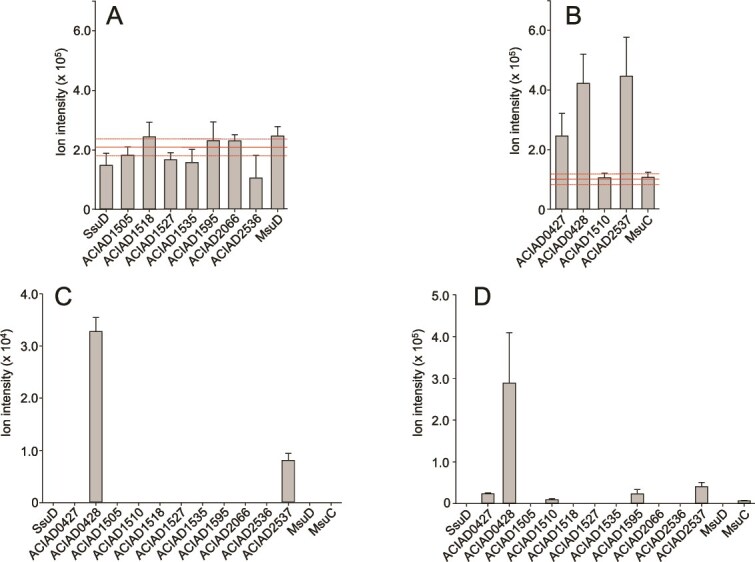
Activity screening of 14 monooxygenases with DMS and DMSO as substrates. Reaction products were analyzed by LC–MS/MS after 5 min of incubation. Results are expressed in arbitrary units (chromatographic peak intensities; *n* = 3). Solid and dashed lines indicate the mean and SD, respectively, of DMSO formation observed in control reactions with MsuE alone (*n* = 3). Monooxygenase concentration was fixed at 0.2 μM in all assays. MsuE concentration was 0.6 μM for group C enzymes and 0.2 μM for group D enzymes. (A) DMSO formation from DMS by group C enzymes. (B) DMSO formation from DMS by group D enzymes. (C) DMSO_2_ formation from DMS. (D) DMSO_2_ formation from DMSO.

No group C enzyme exhibited detectable DMS oxidation (Fig. 4A). Three group D enzymes (ACIAD0427, ACIAD0428, and ACIAD2537) showed significant activity for the conversion of DMS to DMSO (Fig. 4B), with ACIAD0428 and ACIAD2537 also forming DMSO_2_, which confirms a two-step oxidation pathway from DMS to DMSO_2_ (Fig. 3C). With DMSO as substrate, ACIAD0428 was the most active, although ACIAD0427, ACIAD1510, ACIAD1595, ACIAD2537, and ACIAD3474 (MsuC) catalyzed DMSO_2_ formation at lower levels (Fig. 4D).

The three primary enzymes—ACIAD0427, ACIAD0428, and ACIAD2-537—were renamed Dms1, Dms2, and Dms3, respectively (Supplementary Table S7). Their specific activities were measured by GC–MS/MS for increased sensitivity. All three were active on both DMS and DMSO (Table 2), with Dms2 showing the highest efficiency.

**Table 2:** Specific activity of the DMS and DMSO oxygenases detected from LC–MS/MS monooxygenase activity screening.

**Enzyme**	**Enzymatic reaction**	
	DMS ➔ DMSO	DMSO ➔ DMSO_2_
	Specific activity (μmol·min^−1^·mg^−1^)	
Dms1	0.095 ± 0.015	0.097 ± 0.012
Dms2	0.468 ± 0.062	0.432 ± 0.097
Dms3	0.043 ± 0.009	0.097 ± 0.010

Experiments were monitored by GC–MS/MS over a 3-min time course with 1-min sampling intervals. Values correspond to the average of three replicates conducted in 25 mM Tris-HCl pH 7.5, 100 mM NaCl, 1 μM FMN, 1 mM NADH, and 500 μM DMS or DMSO.

### Assessing the physiological role of DMS and DMSO monooxygenases via mutant growth phenotypes

**Figure 5:**
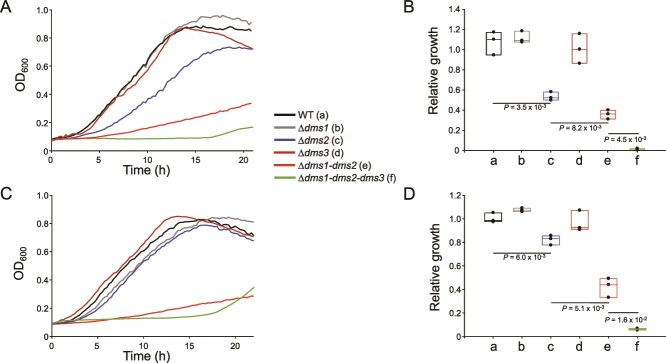
Contribution of individual *Acinetobacter baylyi* ADP1 enzymes to DMS and DMSO utilization. (A) Growth of wild-type and deletion mutants of candidate genes in minimal medium with DMS as the sole sulfur source. (B) Relative growth of mutant strains on DMS compared to sulfate, calculated as the ratio of growth rates on DMS and sulfate. (C) Growth of wild-type and deletion mutants on DMSO as the sole sulfur source. (D) Relative growth of mutant strains on DMSO compared to sulfate. Independent overnight precultures were grown in minimal medium containing SO_4_, harvested and washed before inoculation in minimal medium supplemented with 10 mM of DMS or 2 mM DMSO. Absorbance at 600 nm was monitored using an automated growth curve analysis system (Bioscreen-C; Thermo Fisher Scientific). Data in panels A and C correspond to the average of three biological replicates. Colors indicate the deleted gene(s) in each strain.

To evaluate the physiological relevance of the enzymes active on DMS and DMSO, we analyzed the growth of deletion mutants. Growth on DMS was unaffected in Δ*dms1* and Δ*dms3* (Fig. 5A), which indicates that these genes are not individually essential. In contrast, Δ*dms2* showed a significant growth defect. The Δ*dms1–dms2* double mutant displayed a more pronounced growth defect than the ∆*dms2* mutant, consistent with an additive effect, which was further exacerbated in the ∆*dms1*–*dms2*–*dms3* mutant. Growth on DMS normalized to sulfate confirmed that these defects were specific to DMS metabolism (Fig. 5B). This growth ratio analysis confirmed that Δ*dms1* and Δ*dms3* mutants were not significantly different from the wild type (*P* = .6101 and .5827, respectively), whereas Δ*dms2* showed a decrease (*P* = 3.5 × 10^−3^). Δ*dms1–dms2* exhibited a further reduction in growth rate compared to Δ*dms2* (*P* = 8.2 × 10^−3^), consistent with an additive effect. This growth defect was further pronounced in the Δ*dms1*–*dms2*–*dms3* mutant.

Growth on DMSO as the sulfur source produced similar results. Only the mutants ∆*dms1–dms2* and Δ*dms1*–*dms2*–*dms3* showed a pronounced growth defect (Fig. 5C). Although the growth ratio of Δ*dms1* and Δ*dms3* was unaffected (*P* = 8.87 × 10^−2^ and .5869, respectively), Δ*dms2* displayed slightly reduced growth (*P* = 6.0 × 10^−3^), which was exacerbated in the double mutant compared to Δ*dms2* (*P* = 5.1 × 10^−3^, Fig. 4D). Consistent with the observations made with DMS, growth of the Δ*dms1*–*dms2*–*dms3* triple mutant was nearly abolished.

These observations confirm that Dms1, Dms2, and Dms3 contribute to DMSO_2_ formation, with Dms2 playing the predominant role. Other enzymes, such as ACIAD1510, ACIAD1595, and MsuC, also contribute partially to this pathway (Fig. 4).

### Metabolomic profiling of mutants impaired in DMS metabolism

**Figure 6:**
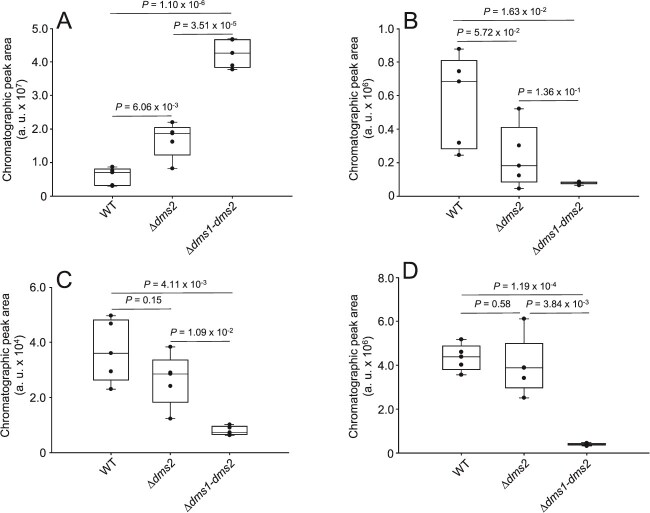
Targeted metabolomic analysis of DMS-derived catabolites in *Acinetobacter baylyi* ADP1 mutants lacking genes involved in DMS oxidation. (A) DMSO; (B) DMSO_2_; (C) MSI; and (D) MSA. Strains were grown in minimal medium containing DMS as the sole sulfur source, and metabolites were quantified from five independent cultures per strain. LC–MS/MS analyses were performed on a QTRAP 5500 mass spectrometer using a MRM-based approach. DMSO and DMSO_2_ were detected in the positive ionization mode; MSI and MSA were detected in the negative ionization mode. The monitored mass transitions were: DMSO, m/z 78.7 → 64.0; DMSO_2_, m/z 94.9 → 62.9; MSI, m/z 78.9 → 64.0; and MSA, m/z 95.0 → 80.0. Values correspond to automated peak integration. *P-*values were determined by Welch’s *t*-test.

To link the growth defects of mutants to DMS metabolism, we performed metabolomic analyses (Fig. 6). The Δ*dms2* mutant accumulated DMSO compared with the wild type (*P* = 6.06 × 10^−3^; Fig. 6A) and this accumulation was higher in the double mutant (*P* = 3.51 × 10^−5^), indicating an additive effect. Conversely, downstream metabolites DMSO_2_, MSI, and MSA decreased in Δ*dms2* (Fig. 6B–D), and more strongly in the double mutant, with significant decreases in DMSO_2_ (*P* = 1.63 × 10^−2^; Fig. 6B), MSI (*P* = 4.11 × 10^−3^; Fig. 6C), and MSA (*P* = 1.19 × 10^−4^; Fig. 6D). This suggests that DMSO oxidation to DMSO_2_ is more limiting than DMS oxidation to DMSO. The *in vivo* metabolite patterns are consistent with the *in vitro* activities of Dms1 and Dms2 (Fig. 4 and Table 2). Deletion of both enzymes substantially reduced DMSO flux, which explains the pronounced growth delay in the double mutant (Fig. 5).

### Kinetic analysis of monooxygenases involved in DMS metabolism

**Figure 7:**
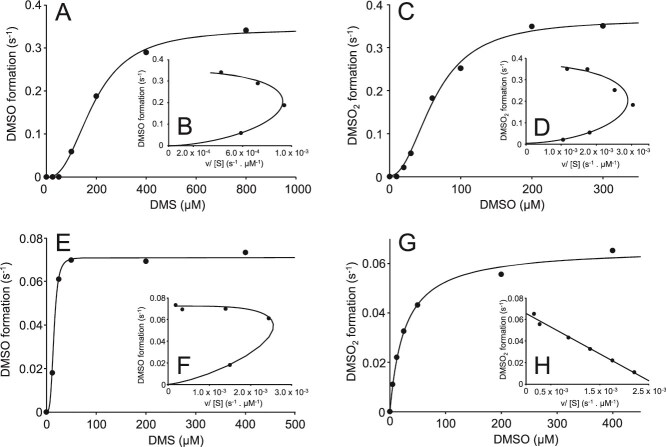
Steady-state kinetics of DMS and DMSO oxidation by Dms2 and Dms1. (A and B) Rates of DMSO formation by Dms2 in the presence of saturating concentrations of DMS. (C and D) Rates of DMSO_2_ formation by Dms2 in the presence of saturating concentrations of DMSO. (E and F) Rates of DMSO formation by Dms1 in the presence of saturating concentrations of DMS. (G and H) Rates of DMSO_2_ formation by Dms1 in the presence of saturating concentrations of DMSO. Data in A, C, and E were fitted using the Hill model v = (*V*_max_  *S*^n^)/(*S*_50_^n^ + *S*^n^). *S*_50_ is the substrate concentration showing half-maximal velocity, *n* is the Hill coefficient, and V_max_ is the maximal velocity. Data in G were fitted using the Michaelis–Menten model. Insets B, D, F, and H show the Eadie–Hofstee representation (v versus v/S) of the kinetics. All data were obtained by GC-–MS/MS. Values correspond to the average of three replicates.

Enzymes converting DMS to sulfite were kinetically characterized. Dms2 showed apparent cooperativity with DMS, consistent with the nonlinear Eadie–Hofstee plots (Fig. 7A and B). However, this apparent cooperativity may result from product inhibition: DMS inhibited DMSO oxidation to DMSO_2_, as evidenced by the decrease in DMSO_2_ formation with increasing DMS concentrations (Supplementary Fig. S3). This inhibition could promote DMSO accumulation at intermediate DMS concentrations and amplify the cooperative pattern. Thus, the kinetic parameters obtained for DMSO formation likely represent apparent values (Table 3). Kinetics with DMSO as substrate also revealed cooperative behavior (Fig. 7C and D), which suggests substrate-induced conformational changes and indicates that Dms2 can function as a truly cooperative enzyme.

**Table 3:** Kinetic parameters of DMS and DMSO oxidation determined from a sigmoidal *V*_max_ model.

Enzyme	Substrate	*V*_max_ (s ^ −1^ )	*S*_50_ (μM)	*n*
Dms2	DMS	0.350 ± 0.010	190.26 ± 8.77	2.49 ± 0.23
	DMSO	0.370 ± 0.010	64.08 ± 3.59	2.21 ± 0.21
Dms1	DMS	0.071 ± 0.001	15.80 ± 0.48	3.92 ± 0.34
Dms3	DMS	0.046 ± 0.003	32.65 ± 3.85	2.62 ± 0.68
	DMSO	0.098 ± 0.002	12.39 ± 0.77	2.24 ± 0.29

Values correspond to the average of three replicates.

Dms1 displayed similar cooperativity with DMS (Fig. 7E and F), but showed hyperbolic kinetics with DMSO (Fig. 7G and H), which suggests that cooperativity arises mainly from DMS inhibition of the second oxidation step. Dms3 exhibited kinetics resembling Dms2, showing cooperative behavior for both DMS and DMSO oxidations (Supplementary Fig. S4). DMS inhibited DMSO_2_ production only above 50 μM.

These results indicate that Dms2 and Dms3 contribute to cooperative control of DMS oxidation, whereas Dms1 cooperativity likely reflects substrate inhibition (Tables 4 and 5). The apparent kinetic parameters for DMSO formation probably underestimate the intrinsic enzyme behavior due to overlap of sequential reactions. MsuD, MsuC, and SsuD were also characterized to complete the kinetic analysis of the full pathway (Supplementary Fig. S5 and Table 4).

**Table 4:** Kinetic properties of Dms1, MsuD, MsuC, and SsuD determined from a hyperbolic *V*_max_ model.

Enzyme	Substrate	*k*_cat_ (s ^ −1^ )	*K*_m_ (μM)	*K*_i_ (μM)	*k*_cat_ /*K*_m_ (s ^ −1^ M ^ −1^ )
Dms1	DMSO	0.067 ± 0.002	26.39 ± 2.58	/	2.5 × 10^3^
MsuD	DMSO_2_	0.090 ± 0.010	5.52 ± 1.57	198.05 ± 59.09	1.62 × 10^4^
MsuC	MSI	0.006 ± 0.001	26.23 ± 4.64	/	2.17 × 10^2^
SsuD	MSA	0.630 ± 0.035	49.75 ± 14.65	/	1.26 × 10^4^

Values correspond to the average of three replicates.

**Table 5:** Comparison of the transcriptional levels of the genes of the DMS assimilation pathway by quantitative PCR following reverse transcription.

Gene	SO_4_ ΔCt	DMS ΔCt	ΔΔCt	2 ^ −ΔΔCt^ (FC)	*P*-value
*msuE*	9.86 ± 0.64	−1.91 ± 0.64	−11.77	3484.33	2.27 × 10^−5^
*dms1*	8.09 ± 0.27	−2.15 ± 0.33	−10.24	1210.73	3.21 × 10^−6^
*dms2*	6.45 ± 0.28	−2.50 ± 0.50	−8.94	491.71	7.88 × 10^−5^
*dms3*	9.56 ± 0.36	0.90 ± 0.25	−8.66	405.44	1.49 × 10^−5^
*msuD*	8.40 ± 0.41	−2.67 ± 0.33	−11.06	2139.91	5.27 × 10^−6^
*ssuD*	9.51 ± 0.31	−2.39 ± 0.30	−11.91	3834.97	1.15 × 10^−6^

FC: fold change. ΔCt values represent mean ± SD (*n* = 3 biological replicates), normalized to *rpoB*. ΔΔCt indicates the difference in mean ΔCt between the two groups. The mean Ct for *rpoB* was 24.67 ± 0.36 (SO_4_) and 26.12 ± 0.47 (DMS). Statistical significance (*P*-value) was calculated using a Welch *t-*test on ΔCt values.

### Transcriptional regulation of the DMS assimilation pathway in response to sulfur availability

Under sulfate-limited conditions, bacteria can synthesize a set of sulfate starvation-induced (SSI) proteins [70–72]. They consist of enzymes and transport systems that enable the uptake and metabolism of alternative sulfur sources from the environment [73]. Based on this framework, we hypothesized that genes involved in DMS-derived sulfur assimilation would be regulated in response to sulfur availability. To test this, we quantified the transcriptional response of six representative genes (*msuE, dms1, dms2, dms3, msuD,* and *ssuD*), using *rpoB* as the normalization gene [74]. Although a slight shift in *rpoB* expression was observed between conditions (mean Ct of 24.67 ± 0.36 for growth with SO_4_ versus 26.12 ± 0.47 for growth with DMS), this 1.45 cycle variation is minimal compared to the huge induction observed for the target genes (from −8.66 to −11.91 ΔΔCt; Table 5), confirming that the magnitude of expression for the target genes far outweighs the minor fluctuations in the reference gene, ensuring the robustness of the data. Altogether, these results indicate that the DMS assimilation pathway is induced in the absence of sulfate, consistent with a broader SSI-type regulatory response.

### Taxonomy and environmental distribution of DMS-oxidizing organisms

**Figure 8:**
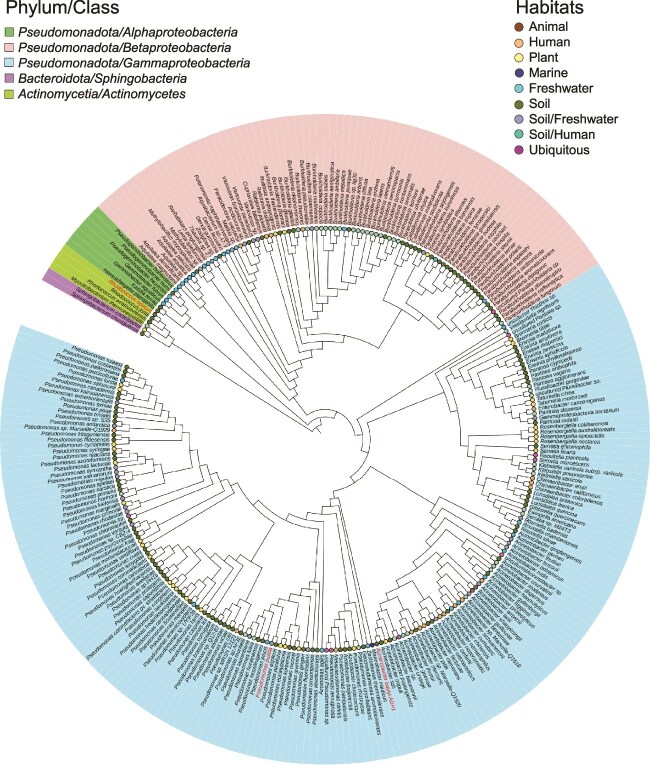
Phylogenetic tree of organisms likely to possess the DMS assimilation pathway. Phylogenetic analysis was performed using MAFFT [49] for multiple sequence alignment, QuickTree [50] for tree building, and iTOL [51] for tree rendering. *Acinetobacter baylyi* ADP1, *Pseudomonas putida*, and *Rhodococcus opacus* are organisms experimentally demonstrated to use DMS as a sulfur source.

To evaluate the environmental distribution of the identified pathway, we screened publicly available bacterial genomes for homologs of the three key enzymes (Dms2, MsuD, and SsuD) whose gene deletions affected growth (Figs 3 and 5). Nearly 300 organisms were identified in which the three selected genes share >50% identity across >80% of their length (Fig. 8 and Supplementary Table S8). These bacteria predominantly belong to the phylum *Pseudomonadota*, especially within the γ-, β-, and to a lesser extent, α-proteobacteria. The majority of these microorganisms are associated with soil environments (67%), with smaller fractions linked to plants (11%) and freshwater habitats (11%). Only one organism, *Marinobacter nauticus*, is reported from a marine environment. These observations suggest that these genes are mostly absent from marine microorganisms and that this pathway is predominantly associated with terrestrial habitats. To validate this correlation between gene presence and DMS utilization, we first examined the γ-proteobacterium *P. putida*. Although strain DS1 was previously shown to grow on DMS [32], its genome sequence was unavailable. We therefore tested strain KT2440, which harbors homologs of Dms2, MsuD, and SsuD, and confirmed its growth on DMS. Similarly, the actinomycete *Rhodococcus opacus* (DSM 43205) could also use DMS (Supplementary Fig. S6).

### Widespread potential for DMS-derived sulfur assimilation across diverse ecosystems

**Figure 9:**
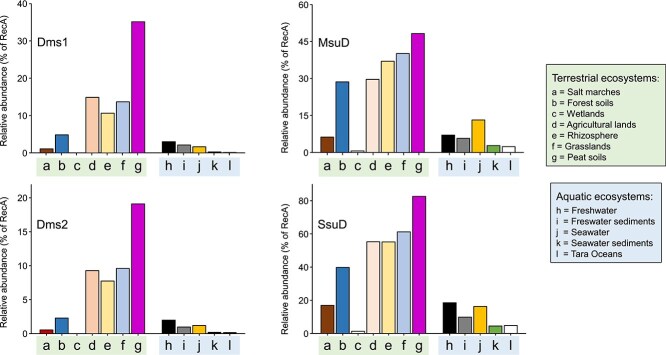
Comparison of normalized values of Dms1, Dms2, MsuD, and SsuD sequences in different environmental metagenomes. Sequences used for mining environmental metagenomic datasets are in Supplementary Table S5, and details of metagenomes are in Supplementary Table S6. The number of unique hits were normalized to the number of unique RecA sequences in each metagenome and are presented as percentage of the total RecA sequences.

The environmental abundance of the genes of the DMS assimilation pathway was estimated by probing metagenomic data sets from various terrestrial and aquatic ecosystems. We focused exclusively on *dms1, dms2, msuD*, and *ssuD*, because several genomes represented in Fig. 8 do not appear to encode *dms3. Pseudomonas putida* and *R. opacus* each harbor a single gene (*PP_2762* and *RMN54_03490*, respectively) that displays homology to both *dms2* and *dms3*, although with higher similarity to *dms2* than to *dms3* (Supplementary Fig. S6). Overall, our analysis indicates that *dms1, dms2, msuD*, and *ssuD* are considerably more abundant in terrestrial habitats than in aquatic ones (Figs 8 and 9). In marine environments, genes specific to DMS utilization (*dms1* and *dms2*) account for <1.6% of genomes (ranging from 0.2% to 1.6%) and in freshwater environments their range from 1 to 3%. In terrestrial habitats, *dms1* and *dms2* are absent from wetlands and only occur at low abundance in salt marshes (<1%), consistent with the anoxic nature of these habitats. These genes are more abundant in agricultural lands, rhizosphere, and grasslands (8%–15%), and can reach 19% and 35% for *dms1* and *dms2,* respectively, in peat soils. The abundance of these genes aligns with the elevated DMS concentrations reported in peatland environments [75–77]. Although peat soils are typically anoxic, projected increases in drought frequency under global warming are likely to enhance peat aeration, particularly in drained systems. Such oxygenation could stimulate the oxidative pathway for DMS assimilation. Across terrestrial habitats, downstream genes involved in DMSO_2_ assimilation (*msuD* and *ssuD*) are generally more abundant than *dms1* and *dms2*. This pattern likely reflects the broader environmental availability of oxidized organosulfur compounds compared with DMS itself.

## Discussion

This study demonstrates that ADP1 utilizes DMS as a sulfur source via a pathway similar to that described in *Pseudomonas* species. Previous models proposed the initial pathway steps but lacked genetic evidence of specific enzymes for these reactions. By identifying the corresponding genes and characterizing all the enzymes of this metabolic route, we provide a complete description of sulfur utilization from DMS in a terrestrial bacterium. The pathway consists of sequential two-component monooxygenases that oxidize DMS to DMSO, then to DMSO_2_, and ultimately to sulfite, which is reduced to hydrogen sulfide for cysteine biosynthesis. Two key features characterize this pathway: functional redundancy, with multiple monooxygenases catalyzing overlapping steps, and substrate promiscuity, with individual enzymes acting on structurally related substrates.

Kinetic characterization revealed diverse enzyme behaviors. Dms1, Dms2, and Dms3 displayed cooperative kinetics, whereas MsuD showed substrate inhibition. In contrast, MsuC and SsuD followed classical Michaelis–Menten kinetics. Catalytic efficiencies varied widely; e.g. the turnover number of MsuC was nearly two orders of magnitude lower than SsuD_ADP1_. Although MsuC can oxidize MSI [38], its low *k*_cat_ and weak growth phenotype (Fig. 3B) suggest this enzyme preferentially targets other organosulfur substrates. Its (low) effect on the conversion of DMSO to DMSO_2_ further supports this hypothesis (Fig. 4D).

MsuD is essential for DMS assimilation but shows modest catalytic efficiency (1.6 × 10^4^ M^−1^ s^−1^), which suggests either limited *in vivo* flux or suboptimal *in vitro* assay conditions. However, the *k*_cat_ determined here for SsuD_ADP1_ (0.6 s^−1^, Table 4) is consistent with previously reported values for SsuD from *E. coli* or MsuD from *P. aeruginosa* (1.5 and 0.3 s^−1^, respectively [47, 67]), which indicate that these modest activities reflect intrinsic enzyme properties. Low turnover rates could be compensated *in vivo* by high intracellular enzyme abundance, thereby maintaining sufficient flux to support growth on DMS as a sulfur source. qPCR data (Table 5) support this hypothesis.

In two-component monooxygenase systems, the reductase and the oxygenase are often coupled both functionally and genetically. This is exemplified by well-characterized systems such as SsuD with SsuE in *E. coli*, LuxA/B with LuxG in *Photobacterium leiognathi*, ActVA/ActVB in *Streptomyces coelicolor*, EmoB and EmoA in *Chelativorans* BNC1, or HPAH C1 and HPAH C2 in *Acinetobacter baumannii* [59, 62, 78–80]. *Pseudomonas* MsuE appears to deviate from this paradigm, because it powers at least three monooxygenases (SfnG, MsuC, and MsuD) involved in the oxidation of DMSO_2_ to sulfite [58]. Our findings expand the repertoire of monooxygenases powered by MsuE. We show in this study that MsuE_ADP1_ supplies electrons to all monooxygenases within the DMS catabolic pathway: Dms1, Dms2, Dms3, MsuD, MsuC, SsuD, and also ACIAD1510 and ACIAD1595 (Fig. 4D). This broad specificity suggests a central metabolic role for MsuE in sulfur assimilation. The substrate promiscuity observed in this study suggests that the catalytic potential of MsuE-dependent monooxygenases is likely underestimated, and that these enzymes may act on a broader range of sulfur-containing substrates. Further work will be required to define this repertoire.

From an evolutionary perspective, using a single reductase may minimize gene duplication and reduce metabolic costs, particularly in sulfur-limited environments. This configuration could enable greater metabolic flexibility: the acquisition of a new monooxygenase gene would be sufficient to expand the range of sulfur compounds an organism can assimilate, without requiring additional reductase machinery. It is plausible that MsuE initially evolved in association with a single monooxygenase, and subsequently broadened its specificity, or alternatively, that multiple monooxygenases independently evolved the capacity to utilize MsuE-derived FMNH_2_.

The presence of an active DMS degradation pathway in soil bacteria highlights the importance of terrestrial DMS metabolism, and suggests that soils may play a significant role in the global sulfur cycle. Atmospheric DMS is rapidly oxidized before deposition [4, 81, 82]. Thus, a specific DMS catabolic pathway in soil bacteria likely reflects endogenous production of DMS in terrestrial environments, rather than uptake from marine emissions.

Terrestrial DMS production remains poorly quantified and has been largely overlooked in global sulfur cycle models. However, several studies have demonstrated DMS production in soil and plant-associated environments [16, 77, 83–85]. Moreover, a recent report [86] indicates that DMSP accumulation occurs universally in plants, although at varying levels, and suggests that terrestrial ecosystems may represent a non-negligible source of DMSP. Microorganisms residing in these environments would be expected to metabolize DMSP and produce DMS, which may serve as a sulfur source for other organisms.

Our analysis revealed that genes associated with DMS-derived sulfur assimilation are widespread across terrestrial environments, with particularly high abundance in peat soils. Peatlands represent a major global carbon reservoir, storing 450–550 Gt of carbon, roughly half of the atmospheric carbon pool [87]. Several of the peat soil metagenomes examined here originate from the “Peat permafrost microbial communities from Stordalen Mire, Sweden” (Supplementary Table S6), a region where the permafrost is thawing rapidly due to global warming [88]. Permafrost covers 15%–22% of the northern hemisphere’s terrestrial surface and stores approximately one-third (~185 Gt) of the carbon in permafrost [89, 90]. The high abundance of DMS-related genes in peat soils is consistent with its substantial functional potential [91]. Episodic oxygenation associated with climate-driven drying may enhance oxidative microbial processes, making aerobic DMS turnover a potentially significant component of sulfur cycling in these systems.

In conclusion, integrating metabolomics, mutant phenotyping, and enzymology allowed the identification and functional characterization of a complete DMS catabolic pathway in a terrestrial bacterium. The presence of this pathway across diverse soil-, freshwater-, and plant-associated taxa indicates that DMS metabolism is widespread in terrestrial ecosystems, representing an important, previously overlooked aspect of the global sulfur cycle.

## Supplementary Material

Supplementary_material_wrag149

## Data Availability

All data generated or analyzed during this study are included in this published article and its Supplementary Information files. Additional source data are available from the corresponding author upon reasonable request.
