# Supplementary material for: Elucidation of dimethyl sulfide assimilation in soil bacteria expands the enzymatic landscape of terrestrial sulfur cycling
Source: ISME J. 2026 Jun 13;20(1):wrag149. doi: 10.1093/ismejo/wrag149 (PMC13352529; doi:10.1093/ismejo/wrag149)
Supplement: Supplementary_material_wrag149 [file supplementary_material_wrag149.zip › Yousfi et al-SI.docx]

**Elucidation of Dimethyl Sulfide Assimilation in Soil Bacteria Expands the Enzymatic Landscape of Terrestrial Sulfur Cycling**

**Short title: Enzymes for DMS Metabolism**

**Yasmeen Yousfi^1^**, **Emilie Pateau^1^**, **Nadia Perchat^1^**, **Jean-Louis Petit^1^**, **Murielle Jérôme^2^,** **William Buchmann^2^**, **Ekaterina Darii^1^**, and **Alain Perret^1^**

^1^Génomique Métabolique, Genoscope, Institut François Jacob, CEA, CNRS, Univ Evry, Université Paris-Saclay, 91057 Evry, France

^2^Université Paris- Saclay, Univ Evry, CY Cergy Paris Université, CNRS, LAMBE, Evry- Courcouronnes, France

***Correspondence :**

Alain Perret

[aperret@genoscope.cns.fr](mailto:aperret@genoscope.cns.fr)

**Supplementary Material**

**This document contains Supplementary Figures 1, 2, 3, 4, 5, 6, and Supplementary Tables 1, 2, 3, 4, 5, 7, and 8.**

**Supplementary Figures**

**
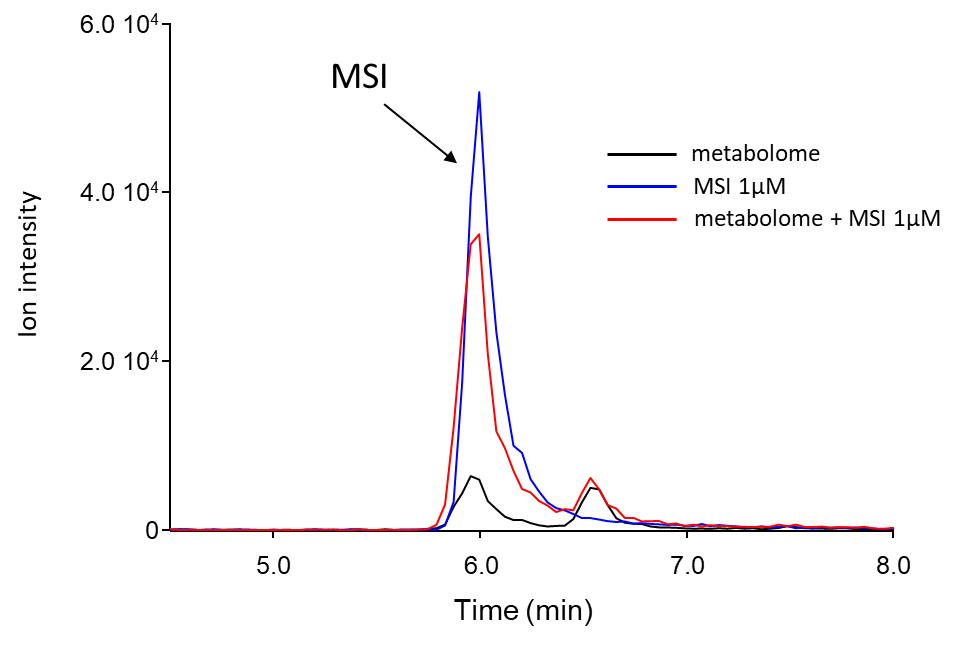
**

**Figure S1.** Detection of MSI in metabolomes of DMS-grown cells of ADP1. MSI was monitored by LC–MS/MS analyses using a multiple reaction monitoring (MRM)–based method. The extracted ion chromatograms are shown for the only MRM transition detectable for MSI (78.9 🡪 64.0). Black, ADP1 wild type metabolome; blue, 1 µM MSI reference standard; red, 1 µM MSI reference standard spiked in wild type metabolome.


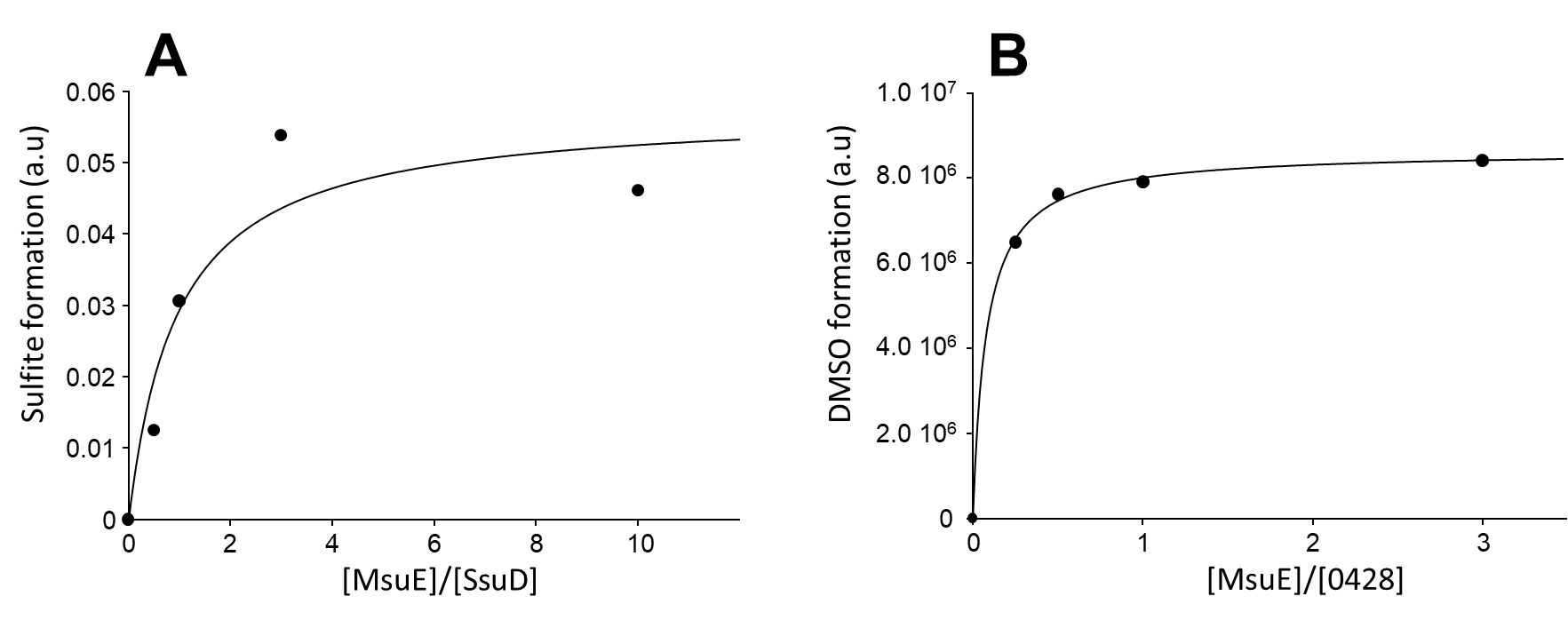


**Figure S2.** Influence of the MsuE-to-monooxygenase molar ratio on catalytic activity.
(A) Effect of the MsuE/SsuD molar ratio on sulfite formation. The concentration of SsuD was kept constant (0.2 µM) while MsuE concentration was varied. (B) Effect of the MsuE/ACIAD0428 molar ratio on DMSO formation. The concentration of ACIAD0428 was kept constant (2 µM) while MsuE concentration was varied. In each case, product formation was monitored every minute for 3 min. Values correspond to the average of three replicates.


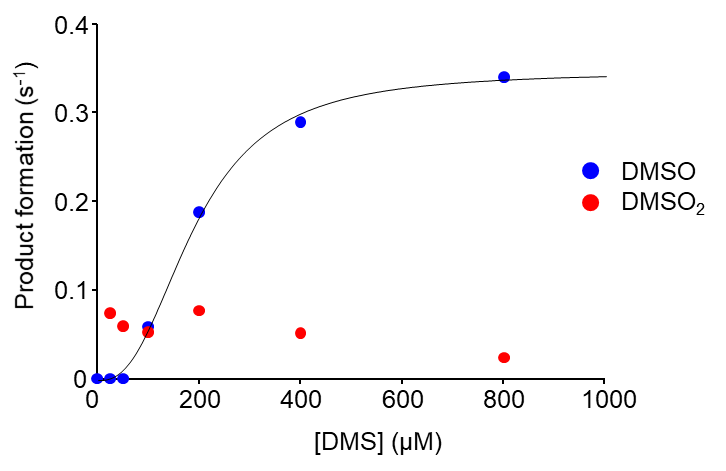


**Figure S3.** Formation rates of DMSO and DMSO₂ catalyzed by Dms2 at increasing DMS concentrations. Data were obtained by GC-MS/MS. Values correspond to the average of three replicates.


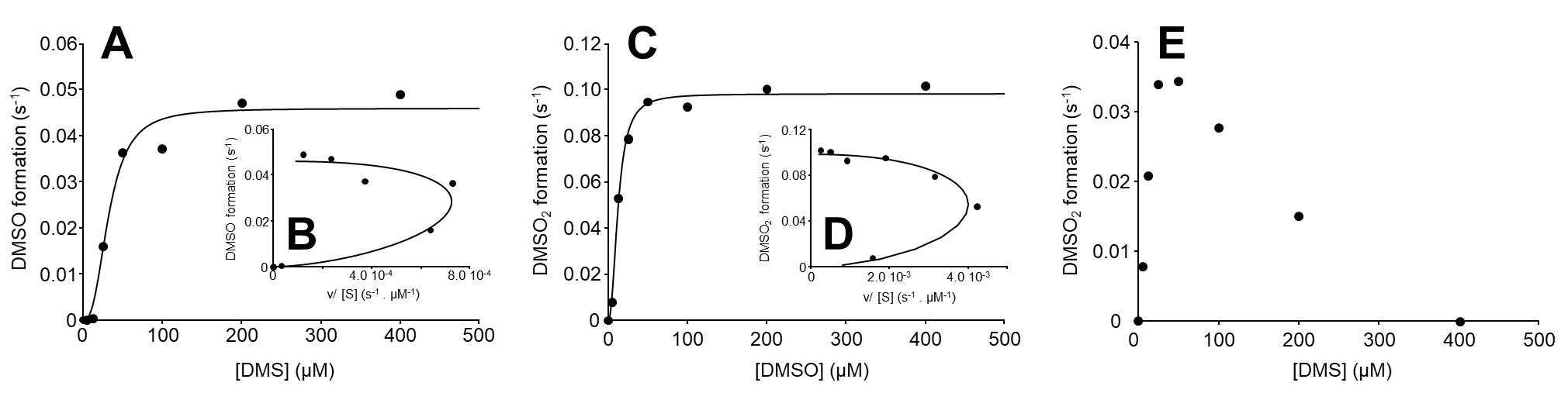


**Figure S4.** Steady-state kinetics of Dms3. (A, B) DMSO formation rate as a function of DMS concentration. (C, D) DMSO_2_ formation rate as a function of DMSO concentration. Data in A and C were fitted using the Hill model *v* = (*V*max *S*^n^)/(*S*_50_^n^ + *S*^n^). *S*_50_ is the substrate concentration showing half-maximal velocity, *n* is the Hill coefficient, and *V*max is the maximal velocity. Insets B and D show the Eadie–Hofstee representation (*v* versus *v*/*S*) of the kinetics. (E) DMSO₂ formation rate of DMSO at increasing DMS concentrations. All data were obtained by GC-MS/MS. Values correspond to the average of three replicates.

**
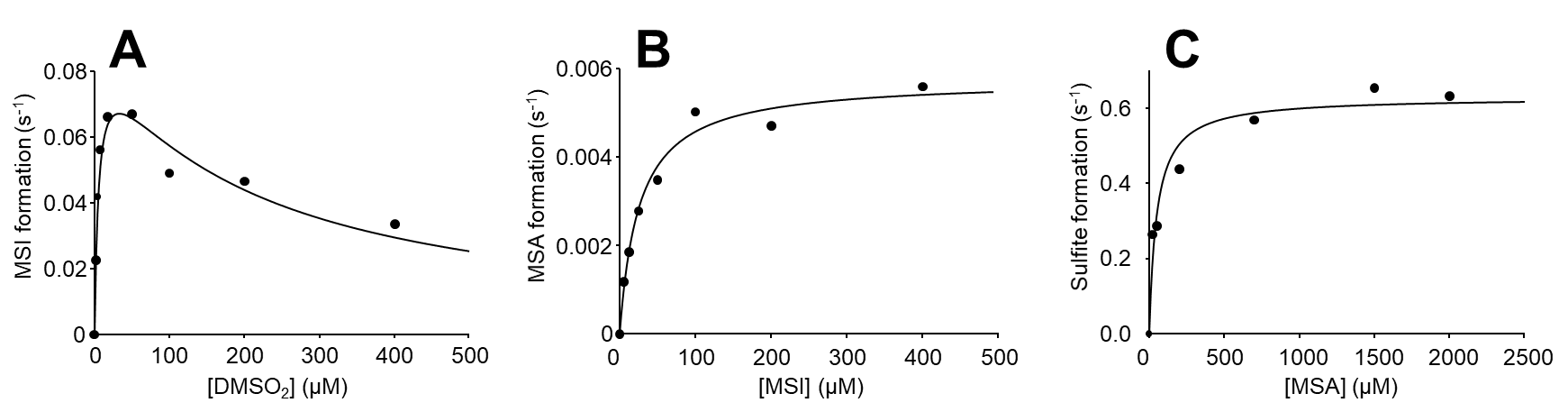
**

**Figure S5.** Steady-state kinetics of MsuD, MsuC and SsuD. (A) MSI formation rate by MsuD as a function of DMSO_2_ concentration. Data were fitted using the substrate inhibition model. (B) MSA formation rate by MsuC as a function of MSI concentration. (C) Sulfite formation rate by SsuD as a function of MSA concentration. Data in (B) and (C) were fitted using the Michaelis–Menten model. All data were obtained by LC-MS/MS. Values correspond to the average of three replicates.

**
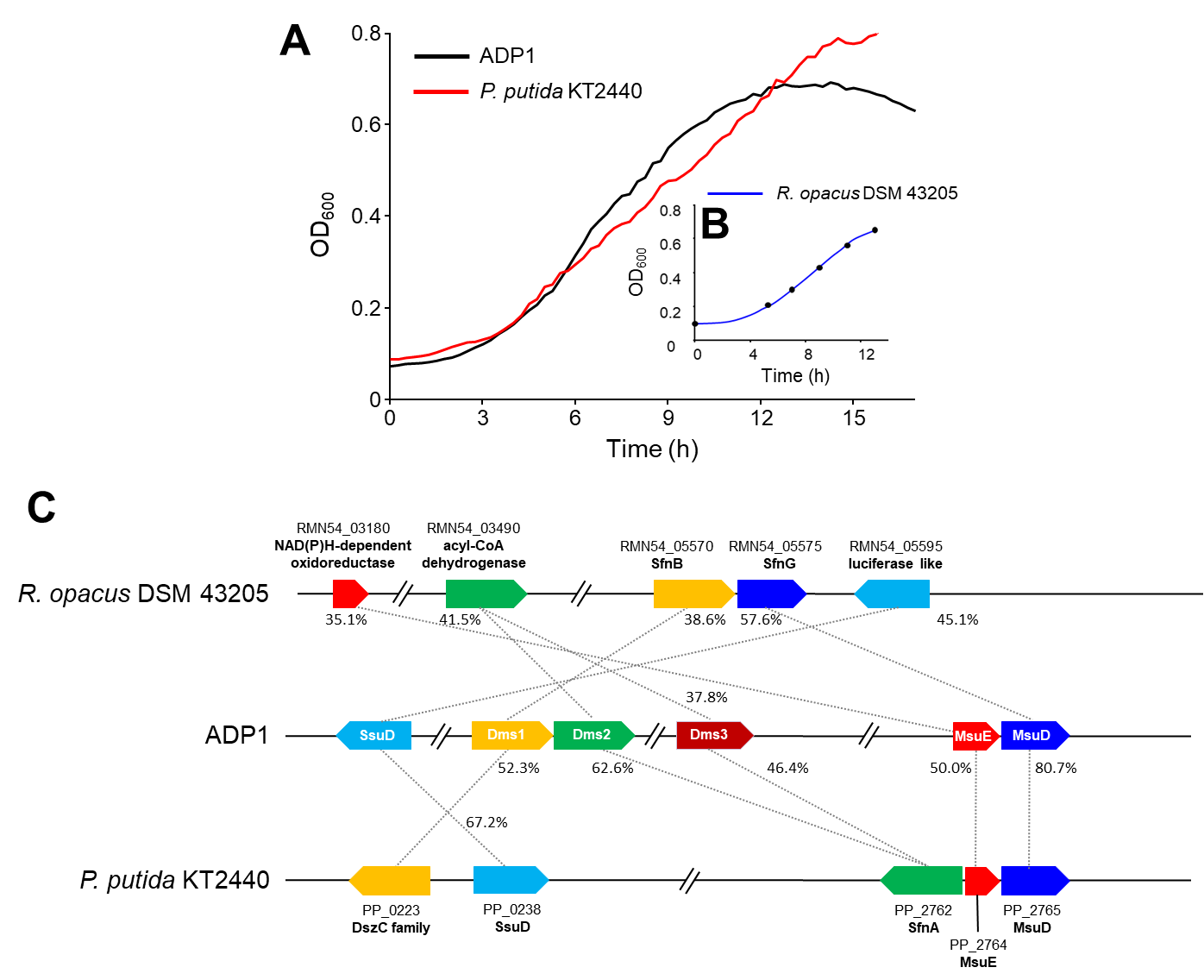
**

**Figure S6**. DMS assimilation in *Pseudomonas putida* KT2440 and *Rhodococcus opacus* DSM 43205. (A) Comparison of wild type ADP1 and *P. putida* growth curves in minimal medium with DMS as the sole sulfur source. Absorbance at 600 nm was monitored using an automated growth curve analysis system (Bioscreen-C; Thermo Fisher Scientific). Data correspond to the average of three biological replicates. (B) Growth of *R. opacus* in minimal medium with DMS as the sole sulfur source. *R. opacus* was cultivated in 50 ml of medium in an INFORS shaking incubator (INFORS-HT, France), supplemented with 0.05% (v/v) Tween 80 to prevent floc formation. Data correspond to the average of two biological replicates. (C) Conservation of the catalytic genes of DMS assimilation in *P. putida* and *R. opacus*. Colocalization of genetic loci was observed through the MicroScope platform. The percentage of identity between the ADP1 sequences and those of *P. putida* and *R. opacus* is indicated along the dotted lines. The symbol “//” means an interruption in the cluster of >10 kb.

**Supplementary Tables**

**Table S1.** MRM transitions of compounds detected by GC-MS/MS. DP, declustering potential; CE, collision energy; CXP, cell exit potential.

| **Compounds** | **Parent mass**  ***m/z*** | **Product mass**  ***m/z*** | **CE**  **Volts** |
| --- | --- | --- | --- |
| DMSO * | 78 | 63 | 12 |
| DMSO † | 63 | 45 | 8 |
| DMSO_2_ * | 94 | 79 | 6 |

*Transitions used for quantification

† In-source fragment of DMSO. Transition *m/z* 63 🡪 45 was used to monitor DMSO

**Table S2**. MRM transitions of compounds detected by LC-MS/MS. DP, declustering potential; CE, collision energy; CXP, cell exit potential.

| **Compounds** | **Parent mass**  ***m/z*** | **Product mass**  ***m/z*** | **DP**  **Volts** | **CE**  **Volts** | **CXP**  **Volts** |
| --- | --- | --- | --- | --- | --- |
| DMSO * | 78.7 | 64.0 | 60 | 21 | 10 |
| DMSO | 78.7 | 60.9 | 60 | 17 | 8 |
| DMSO_2_ * | 94.9 | 62.9 | 60 | 21 | 10 |
| DMSO_2_ | 94.9 | 64.9 | 60 | 33 | 10 |
| MSI * | 78.9 | 64.0 | - 40 | - 14 | - 9 |
| MSI | 78.9 | 61.9 | - 40 | - 12 | - 7 |
| MSA * | 95.0 | 80.0 | - 70 | - 22 | - 7 |
| MSA | 95.0 | 77.0 | - 70 | - 16 | - 7 |

*Transitions used for quantification

**Table S3.** Candidate genes coding for monooxygenases involved in sulfur metabolism in ADP1

| Gene ID | Current annotation (product) | Swissprot similarity | Group |
| --- | --- | --- | --- |
| *ACIAD0036* | Alkanesulfonate monooxygenase (SsuD) | Alkanesulfonate monooxygenase (SsuD) | C |
| *ACIAD0427* | putative acyl-CoA dehydrogenase | DBT monooxygenase (DszC) | D |
| *ACIAD0428* | putative acyl-CoA dehydrogenase | Monooxygenase (SfnC) | D |
| *ACIAD1505* | putative DBT desulfurization enzyme | Alkanesulfonate monooxygenase (SsuD) | C |
| *ACIAD1510* | putative desulfurization enzyme C (DBT) | DBT monooxygenase (dszC) | D |
| *ACIAD1518* | putative sulfonate monooxygenase | Alkanesulfonate monooxygenase (SsuD) | C |
| *ACIAD1527* | putative alkanesulfonate monooxygenase | Alkanesulfonate monooxygenase (SsuD) | C |
| *ACIAD1535* | putative sulfonate monooxygenase | Alkanesulfonate monooxygenase (SsuD) | C |
| *ACIAD1595* | nitrilotriacetate monooxygenase (NTA-MO A) | N-acetyl-S-(2-succino)cysteine monooxygenase | C |
| *ACIAD2064* | putative acyl-CoA dehydrogenase | DBT monooxygenase (dszC) | D |
| *ACIAD2065* | putative acyl-CoA dehydrogenase | DBT monooxygenase (dszC) | D |
| *ACIAD2066* | putative monooxygenase (DszA-like) | DMS monooxygenase (DmoA) | C |
| *ACIAD2536* | putative methylene-H_4_MPT reductase | DMS monooxygenase (DmoA) | C |
| *ACIAD2537* | putative acyl-CoA dehydrogenase | Monooxygenase (SfnC) | D |
| *ACIAD3471* | putative sulfonate monooxygenase (MsuD) | DMSO_2_ monooxygenase | C |
| *ACIAD3474* | putative FMNH2-dependent monooxygenase | Monooxygenase (SfnC) | D |

DBT = Dibenzothiophene

**Table S4**. Oligonucleotide primers used in the construction of Δ*dms1-dms2* and Δ*dms1-dms2-dms3* mutants, and for qPCR analysis.

| Primers | Sequences (5’ 🡪 3’) |
| --- | --- |
| Δdms1-dms2_3 | CTCGCATTCTTTGCCATATTAG |
| Δdms1-dms2_4 | TTTTTATGATTTGAATTGGAGGCTGGGTCTGATCTACCCTCTTGATG |
| Δdms1-dms2_5 | CGATGAGTTTTTCTAAGCATGCGGAGCTGGTATTCTGCTTCAAATCCTTTC |
| Δdms1-dms2_6 | CTACTTTTTGCGATGGCTTATC |
| Δdms1-dms2_7 | TCCACTTGCTGTTGAATTGCG |
| Δdms1-dms2_8  Δdms3_4_Apra  Δdms3_5_Apra  FW_rpoB  RV_rpoB  FW_msuE  RV_msuE  FW_dms1  RV_dms1  FW_dms2  RV_dms2  FW_dms3  RV_dms3  FW_msuD  RV_msuD  FW_ssuD  RV_ssuD | CGGGTGGGAATGTATTATGGAG  TTTTTATGATTTGAATTGGAGGCTGGGAATTCATAATTTTTTTATCATATTTAAAATGTATG  CTCGCCAGTCGATTGGCTAAGCTCATGAGAAAAATATAGAATTAAAATGAATATCATGATC  TCTTCGCGGCTCAACTTATG  CGCCTGGTGAACGATGTAAT  CGCTCAATATTGTGGCTGTATCAG  AAGGCATCCGCCGCTTCGAG  AGGATGGCTTGCCCGTTGCA  CTGCATCAACTGCTGCCTGAAT  AATAGTGTAGGCCAGCGTATTG  TATGGTGCCACTTCCTGTTG  CCTGAATTAGACACCGCAACCATTTAT  CAAATAATTCAGGTATACTCGCCTC  GGCCATGGAAACCTGTTCTA  ATTGTCTTGTGTCCAGATACCC  CTCGAAGTCTCACCGAATCTTT  AAGCGGAAGCAGTGGAAATA |

**Table S5.** Sequences used for mining environmental metagenomic datasets.

| Organism | locus_tag | NCBI  Accession number | Protein |  |
| --- | --- | --- | --- | --- |
| *Acinetobacter baylyi* ADP1 | ACIAD0427 | CAG67369.1 | Dms1 |  |
|  | ACIAD0428 | CAG67370.1 | Dms2 |  |
|  | ACIAD3471 | CAG70120.1 | MsuD |  |
|  | ACIAD0036 | CAG67018.1 | SsuD |  |
|  | ACIAD1385 | CAG68251.1 | RecA |  |
|  |  |  |  |  |
| *Pseudomonas putida* KT2440 | PP_0223 | AAN65855.1 | Dms1 |  |
|  | PP_2762 | AAN68370.1 | Dms2 |  |
|  | PP_2765 | AAN68373.1 | MsuD |  |
|  | PP_0238 | AAN65870.1 | SsuD |  |
|  | PP_1629 | AAN67250.1 | RecA |  |
|  |  |  |  |  |
| *Rhodococcus opacus* DSM 43205 | RMN54_05570 | XPE29868.1 | Dms1 |  |
|  | RMN54_03490 | XPE29476.1 | Dms2 |  |
|  | RMN54_05575 | XPE29869.1 | MsuD |  |
|  | RMN54_05595 | XPE29873.1 | SsuD |  |
|  | RMN54_20665 | XPE25985.1 | RecA |  |

**Table S7**: Function of the genes involved in DMS assimilation.

| Gene ID | Function | Gene name |
| --- | --- | --- |
| *ACIAD0036* | MSA monooxygenase | *ssuD* |
| *ACIAD0427* | DMS monooxygenase | *dms1* |
| *ACIAD0428* | DMS monooxygenase | *dms2* |
| *ACIAD2537* | DMS monooxygenase | *dms3* |
| *ACIAD3470* | Flavin reductase | *msuE* |
| *ACIAD3471* | DMSO_2_ monooxygenase | *msuD* |
| *ACIAD3474* | MSI monooxygenase | *msuC* |

**Table S8.** Candidate Organisms Possessing Homologous Genes of SsuD, Dms2, and MsuD.

| Genome | 16S RNA  GenBank Accession | Genome | 16S RNA  GenBank Accession |
| --- | --- | --- | --- |
| *Acidovorax sp.* | PX136319.1 | *Aquitalea aquatica* | ON013926.1 |
| *Acinetobacter baumannii* | PX210433.1 | *Aquitalea denitrificans* | KJ186936.1 |
| *Acinetobacter amyesii* | ON598632.1 | *Aquitalea magnusonii* | KJ186933.1 |
| *Acinetobacter baylyi ADP1* | Bioproject: PRJNA12352 ACIADrRNA16S_1 | *Aquitalea palustris* | OP902205.1 |
| *Acinetobacter bereziniae* | PQ722333.1 | *Aquitalea sp.* | MT101743.1 |
| *Acinetobacter bohemicus* | PX518672.1 | *Azomonas agilis* | ON209960.1 |
| *Acinetobacter brisouii* | PX206076.1 | *Azotobacter beijerinckii* | PV653209.1 |
| *Acinetobacter calcoaceticus* | PX352510.1 | *Azotobacter chroococcum* | PX270285.1 |
| *Acinetobacter chinensis* | PP218107.1 | *Brenneria corticis* | NR_171515.1 |
| *Acinetobacter courvalinii* | PQ722330.1 | *Brenneria tiliae* | OM505020.1 |
| *Acinetobacter cumulans* | OQ780965.1 | *Burkholderia aenigmatica* | OR044071.1 |
| *Acinetobacter dispersus* | OR865120.1 | *Burkholderia ambifaria* | PV889302.1 |
| *Acinetobacter gerneri* | OP818100.1 | *Burkholderia anthina* | OR437497.1 |
| *Acinetobacter guerrae* | NR_179028.1 | *Burkholderia arboris* | PQ373032.1 |
| *Acinetobacter guillouiae* | PX482853.1 | *Burkholderia catarinensis* | PV490897.1 |
| *Acinetobacter gyllenbergii* | PQ857636.1 | *Burkholderia cenocepacia* | PX062293.1 |
| *Acinetobacter haemolyticus* | PV480881.1 | *Burkholderia cepacia* | PX241365.1 |
| *Acinetobacter higginsii* | OR350513.1 | *Burkholderia contaminans* | PX062276.1 |
| *Acinetobacter ihumii* | NR_179446.1 | *Burkholderia diffusa* | PQ867108.1 |
| *Acinetobacter lactucae* | PV424148.1 | *Burkholderia gladioli* | PV902813.1 |
| *Acinetobacter lanii* | NR_180591.1 | *Burkholderia glumae* | PV760244.1 |
| *Acinetobacter modestus* | PV645882.1 | *Burkholderia lata* | PP767697.1 |
| *Acinetobacter nematophilus* | PX204969.1 | *Burkholderia latens* | MW485434.1 |
| *Acinetobacter nosocomialis* | PQ435189.1 | *Burkholderia mayonis* | NR_178588.1 |
| *Acinetobacter oleivorans* | PQ782802.1 | *Burkholderia metallica* | PV889261.1 |
| *Acinetobacter pittii* | PX365909.1 | *Burkholderia multivorans* | PV687737.1 |
| *Acinetobacter populi* | NR_145864.1 | *Burkholderia oklahomensis* | MT319078.1 |
| *Acinetobacter proteolyticus* | PQ722335.1 | *Burkholderia plantarii* | PQ451578.1 |
| *Acinetobacter puyangensis* | NR_109507.1 | *Burkholderia pseudomallei* | PX241974.1 |
| *Acinetobacter qingfengensis* | MZ203625.1 | *Burkholderia puraquae* | PQ657140.1 |
| *Acinetobacter rudis* | PX247670.1 | *Burkholderia pyrrocinia* | PX226121.1 |
| *Acinetobacter seifertii* | PP086587.1 | *Burkholderia seminalis* | PQ782529.1 |
| *Acinetobacter shaoyimingii* | NR_181115.1 | *Burkholderia sp. lig30* | JQ917972.1 |
| *Acinetobacter sichuanensis* | PV478488.1 | *Burkholderia stabilis* | PV424058.1 |
| *Acinetobacter soli* | PX498014.1 | *Burkholderia territorii* | PV720019.1 |
| *Acinetobacter sp.* | PX500807.1 | *Burkholderia thailandensis* | PX225948.1 |
| *Acinetobacter sp. Marseille-Q1618* | LR746132.1 | *Burkholderia vietnamiensis* | PV361866.1 |
| *Acinetobacter sp. Marseille-Q1620* | LR782183.1 | *Caballeronia choica* | OP935848.1 |
| *Acinetobacter stercoris* | NR_181117.1 | *Caballeronia mineralivorans* | PV784153.1 |
| *Acinetobacter thutiue* | PP927007.1 | *Caballeronia sordidicola* | OM670232.1 |
| *Acinetobacter ursingii* | PV953629.1 | *Chimaeribacter arupi* | NR_180435.1 |
| *Aquabacterium* sp. A7-Y | KF135219.1 | *Chimaeribacter californicus* | NR_180433.1 |

| *Chimaeribacter coloradensis* | NR_180434.1 | *Paraburkholderia dipogonis* | MZ234651.1 |
| --- | --- | --- | --- |
| *Cupriavidus basilensis* | ON408506.1 | *Paraburkholderia eburnea* | OP986475.1 |
| *Derxia gummosa* | NR_114127.1 | *Paraburkholderia ferrariae* | OP986707.1 |
| *Derxia lacustris* | NR_108927.1 | *Paraburkholderia fungorum* | PX248578.1 |
| *Enterobacter cancerogenus* | PV715764.1 | *Paraburkholderia guartelaensis* | MK690540.1 |
| *Erwinia amylovora* | PX106820.1 | *Paraburkholderia hospita* | PQ060375.1 |
| *Erwinia aphidicola* | PV951594.1 | *Paraburkholderia kururiensis* | PV760335.1 |
| *Erwinia mallotivora* | PQ670069.1 | *Paraburkholderia lycopersici* | NR_178323.1 |
| *Erwinia persicina* | PV804073.1 | *Paraburkholderia nemoris* | PP469570.1 |
| *Erwinia phyllosphaerae* | NR_181782.1 | *Paraburkholderia nodosa* | LC516071.1 |
| *Erwinia rhapontici* | PV804071.1 | *Paraburkholderia oxyphila* | NR_112888.1 |
| *Ewingella americana* | PV355096.1 | *Paraburkholderia phenoliruptrix* | PV248929.1 |
| *Gammaproteobacteria bacterium* | PQ451780.1 | *Paraburkholderia polaris* | NR_180943.1 |
| *Gemmobacter aquaticus* | NR_116272.1 | *Paraburkholderia sacchari* | PQ722410.1 |
| *Gemmobacter fulvus* | NR_181766.1 | *Paraburkholderia sediminicola* | PV784146.1 |
| *Geopseudomonas aromaticivorans* | OK324373.1 | *Paraburkholderia sejongensis* | MZ031499.1 |
| *Gibbsiella quercinecans* | PQ373180.1 | *Paraburkholderia steynii* | OP847085.1 |
| *Hansschlegelia plantiphila* | MT760665.1 | *Paraburkholderia terrae* | OR536592.1 |
| *Kaistia terrae* | OP804310.1 | *Paraburkholderia tropica* | PP783837.1 |
| *Klebsiella pneumoniae* | PX248379.1 | *Paracidovorax wautersii* | PV599844.1 |
| *Klebsiella variicola* | PX254056.1 | *Pararobbsia silviterrae* | NR_178810.1 |
| *Klebsiella variicola subsp. variicola* | PQ763953.1 | *Pluralibacter gergoviae* | PQ336984.1 |
| *Leeia speluncae* | OK564662.1 | *Polaromonas naphthalenivorans* | PQ217066.1 |
| *Lonsdalea britannica* | MN646990.1 | *Pseudogemmobacter bohemicus* | NR_165010.1 |
| *Lonsdalea iberica* | NR_118126.1 | *Pseudogemmobacter hezensis* | NR_181079.1 |
| *Lonsdalea quercina* | PV951625.1 | *Pseudogemmobacter humi* | NR_171533.1 |
| *Malikia sp.* | OR857043.1 | *Pseudomonas abieticivorans* | ON945574.1 |
| *Marinobacter nauticus* | PV444578.1 | *Pseudomonas aeruginosa* | PX255551.1 |
| *Metapseudomonas otitidis* | PX275544.1 | *Pseudomonas alvandae* | PV803735.1 |
| *Methylobacillus rhizosphaerae* | NR_132610.1 | *Pseudomonas amygdali* | NR_036999.1 |
| *Methylobacillus sp.* | PQ057152.1 | *Pseudomonas antarctica* | PV875671.1 |
| *Mycolicibacterium thermoresistibile* | MW332175.1 | *Pseudomonas arsenicoxydans* | PX062196.1 |
| *Pantoea agglomerans* | PX096444.1 | *Pseudomonas asplenii* | PX023257.1 |
| *Pantoea anthophila* | PV646230.1 | *Pseudomonas avellanae* | PQ520783.1 |
| *Pantoea cypripedii* | PX056924.1 | *Pseudomonas azotoformans* | PV803802.1 |
| *Pantoea dispersa* | PX210431.1 | *Pseudomonas brassicacearum* | NR_024950.1 |
| *Pantoea rodasii* | PX116732.1 | *Pseudomonas canadensis* | PV992060.1 |
| *Pantoea vagans* | PX271198.1 | *Pseudomonas cannabina* | OM827349.1 |
| *Paraburkholderia aromaticivorans* | OP363857.1 | *Pseudomonas caricapapayae* | OR889279.1 |
| *Paraburkholderia aspalathi* | NR_133709.1 | *Pseudomonas caspiana* | PP529725.1 |
| *Paraburkholderia bannensis* | MT101733.1 | *Pseudomonas chlororaphis* | PX069530.1 |
| *Paraburkholderia caffeinilytica* | PP494589.1 | *Pseudomonas citri* | OM327744.1 |

| *Pseudomonas congelans* | PV191096.1 | *Pseudomonas piscis* | PQ782458.1 |
| --- | --- | --- | --- |
| *Pseudomonas coronafaciens* | PQ216325.1 | *Pseudomonas poae* | PX238594.1 |
| *Pseudomonas coronafaciens pv. striafaciens* | MF693348.1 | *Pseudomonas prosekii* | PV927190.1 |
| *Pseudomonas corrugata* | PV687381.1 | *Pseudomonas protegens* | PV770137.1 |
| *Pseudomonas costantinii* | OQ456008.1 | *Pseudomonas psychrophila* | PV803717.1 |
| *Pseudomonas cyclaminis* | LC807668.1 | *Pseudomonas putida KT2440* | PX136557.1 |
| *Pseudomonas eucalypticola* | OQ565619.1 | *Pseudomonas reactans* | PV185945.1 |
| *Pseudomonas extremorientalis* | PV670030.1 | *Pseudomonas rhizoryzae* | NR_180517.1 |
| *Pseudomonas farris* | LC807670.1 | *Pseudomonas rhodesiae* | LC807613.1 |
| *Pseudomonas ficuserectae* | OR016535.1 | *Pseudomonas salomonii* | OL687323.1 |
| *Pseudomonas fildesensis* | PV803746.1 | *Pseudomonas saponiphila* | PX103148.1 |
| *Pseudomonas fluorescens* | PX241972.1 | *Pseudomonas savastanoi* | PP506698.1 |
| *Pseudomonas fragi* | PV803815.1 | *Pseudomonas sessilinigenes* | PP874572.1 |
| *Pseudomonas frederiksbergensis* | PX111488.1 | *Pseudomonas simiae* | PV803796.1 |
| *Pseudomonas fulva* | PX096446.1 | *Pseudomonas sp.* | PX271062.1 |
| *Pseudomonas gingeri* | OQ660461.1 | *Pseudomonas sp. CBZ-4* | JQ782892.1 |
| *Pseudomonas graminis* | PV355175.1 | *Pseudomonas sp. Marseille-Q1929* | LR862420.1 |
| *Pseudomonas grimontii* | PX248456.1 | *Pseudomonas sp. MRSN 12121* | OP353554.1 |
| *Pseudomonas hormoni* | OQ674074.1 | *Pseudomonas sp. Q12-87* | DQ453830.1 |
| *Pseudomonas huanghezhanensis* | NR_199516.1 | *Pseudomonas sp. S35* | KT890311.1 |
| *Pseudomonas hunanensis* | OP954683.1 | *Pseudomonas sp. UYIF39* | KP744606.2 |
| *Pseudomonas kairouanensis* | PV653642.1 | *Pseudomonas sp.AF32* | EU680973.2 |
| *Pseudomonas karstica* | PV094573.1 | *Pseudomonas sp.PH1b* | KF557601.1 |
| *Pseudomonas kielensis* | PV739509.1 | *Pseudomonas spelaei* | PQ520722.1 |
| *Pseudomonas kitaguniensis* | PQ215998.1 | *Pseudomonas synxantha* | PV803838.1 |
| *Pseudomonas koreensis* | PX232766.1 | *Pseudomonas syringae* | PX242667.1 |
| *Pseudomonas lactucae* | PQ870076.1 | *Pseudomonas tolaasii* | PX063865.1 |
| *Pseudomonas laurentiana* | PQ001949.1 | *Pseudomonas trivialis* | PV653645.1 |
| *Pseudomonas lini* | OP954668.1 | *Pseudomonas umsongensis* | PX102336.1 |
| *Pseudomonas lurida* | PV803737.1 | *Pseudomonas versuta* | PV803809.1 |
| *Pseudomonas mandelii* | PX062195.1 | *Pseudomonas viciae* | PP795042.1 |
| *Pseudomonas marginalis* | PV803706.1 | *Pseudomonas viridiflava* | LC807625.1 |
| *Pseudomonas migulae* | PV636728.1 | *Pseudomonas yamanorum* | PV803728.1 |
| *Pseudomonas monteilii* | PV489130.1 | *Ralstonia insidiosa* | PX062041.1 |
| *Pseudomonas moorei* | PQ782730.1 | *Ralstonia pickettii* | PX273899.1 |
| *Pseudomonas morbosilactucae* | LC659975.1 | *Raoultella planticola* | PQ684024.1 |
| *Pseudomonas oryzihabitans* | OP210297.1 | *Rhodococcus jostii* | OR215166.1 |
| *Pseudomonas ovata* | PQ756898.1 | *Rhodococcus opacus* | PP892107.1 |
| *Pseudomonas palleroniana* | PP758412.1 | *Rhodococcus wratislaviensis* | PV988470.1 |
| *Pseudomonas panipatensis* | OR569089.1 | *Rivihabitans pingtungensis* | NR_133846.1 |
| *Pseudomonas parafulva* | PX232808.1 | *Robbsia andropogonis* | PV759631.1 |
| *Pseudomonas pisciculturae* | PX055714.1 | *Rosenbergiella australiborealis* | MT544582.1 |

| *Rosenbergiella collisarenosi* | MT544583.1 | *Thauera sp. 27* | AY838760.1 |
| --- | --- | --- | --- |
| *Rosenbergiella epipactidis* | PV951623.1 | *Trinickia symbiotica* | NR_178463.1 |
| *Rosenbergiella nectarea* | PV052756.1 | *uncultured Acinetobacter sp.* | PP506771.1 |
| *Rouxiella badensis* | ON626777.1 | *uncultured Pantoea sp.* | PP469567.1 |
| *Rouxiella chamberiensis* | PP783845.1 | *uncultured Paraburkholderia sp.* | PP780967.1 |
| *Rouxiella silvae* | PP851152.1 | *uncultured Pluralibacter sp.* | MW494886.1 |
| *Serratia entomophila* | OR826282.1 | *uncultured Pseudomonas sp.* | PX064094.1 |
| *Serratia ficaria* | PV951592.1 | *uncultured Thiothrix sp.* | PP946765.1 |
| *Serratia marcescens* | PX248434.1 | *Variovorax boronicumulans* | PX111475.1 |
| *Serratia sp. M24T3* | HQ538811.2 | *Variovorax paradoxus* | PX111472.1 |
| *Sphingobacterium chungjuense* | NR_179061.1 | *Variovorax sp.* | PX238593.1 |
| *Sphingobacterium corticibacter* | NR_175481.1 | *Vreelandella nigrificans* | PV335932.1 |
| *Tatumella citrea* | OP890299.1 | *Zestomonas thermotolerans* | PX056837.1 |
| *Tatumella morbirosei* | OR821299.1 | *Zoogloea sp.* | PQ282665.1 |
